# Generative Artificial Intelligence for Designing Multi-Scale Hydrogen Fuel Cell Catalyst Layer Nanostructures

**DOI:** 10.1021/acsnano.4c04943

**Published:** 2024-07-10

**Authors:** Zhiqiang Niu, Wanhui Zhao, Hao Deng, Lu Tian, Valerie J. Pinfield, Pingwen Ming, Yun Wang

**Affiliations:** † Renewable Energy Resources Lab, Department of Mechanical and Aerospace Engineering, The University of California, Irvine, California 92697, United States; ‡ Department of Aeronautical and Automotive Engineering, Loughborough University, Loughborough LE11 3TU, U.K.; § Clean Energy Automotive Engineering Centre, School of Automotive Studies, Tongji University, Shanghai 201804, China; ∥ Department of Chemical Engineering, Loughborough University, Loughborough LE11 3TU, U.K.; ⊥ Shanghai Hydrogen Propulsion Technology Company Limited, Shanghai 201800, China; # College of Aeronautical Engineering, Civil Aviation University of China, Tianjin 300300, China

**Keywords:** fuel cells, generative artificial intelligence, multiscale design, multiphysics, catalyst layer

## Abstract

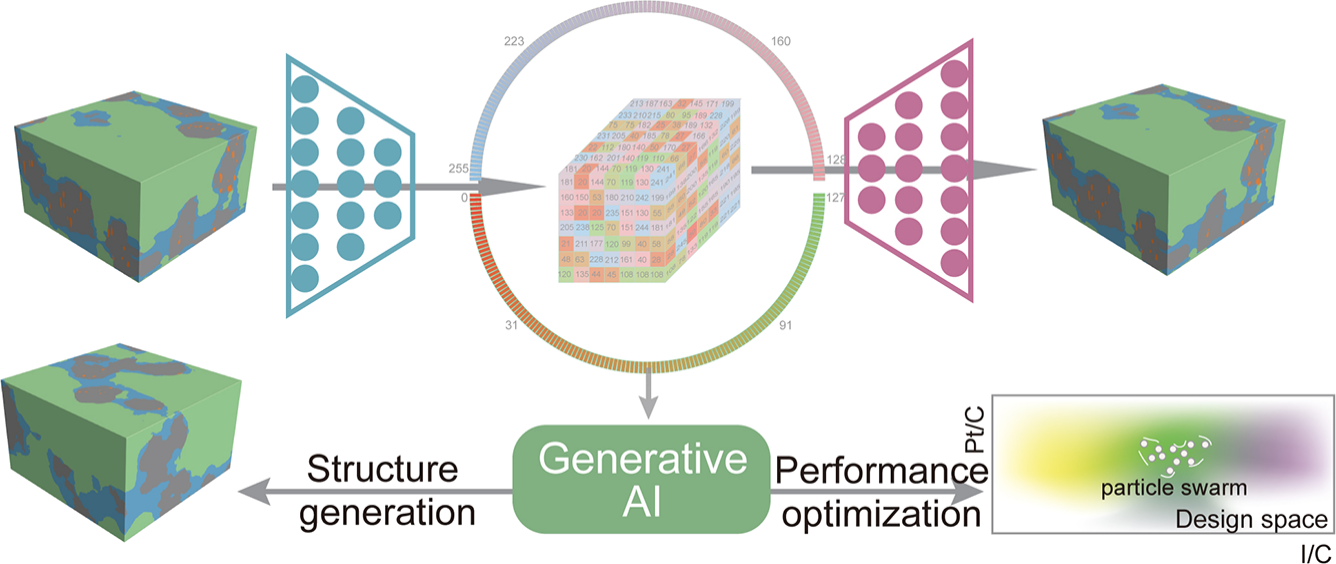

Multiscale design of catalyst layers (CLs) is important to advancing hydrogen electrochemical conversion devices toward commercialized deployment, which has nevertheless been greatly hampered by the complex interplay among multiscale CL components, high synthesis cost and vast design space. We lack rational design and optimization techniques that can accurately reflect the nanostructure-performance relationship and cost-effectively search the design space. Here, we fill this gap with a deep generative artificial intelligence (AI) framework, GLIDER, that integrates recent generative AI, data-driven surrogate techniques and collective intelligence to efficiently search the optimal CL nanostructures driven by their electrochemical performance. GLIDER achieves realistic multiscale CL digital generation by leveraging the dimensionality-reduction ability of quantized vector-variational autoencoder. The powerful generative capability of GLIDER allows the efficient search of the optimal design parameters for the Pt-carbon-ionomer nanostructures of CLs. We also demonstrate that GLIDER is transferable to other fuel cell electrode microstructure generation, *e.g*., fibrous gas diffusion layers and solid oxide fuel cell anode. GLIDER is of potential as a digital tool for the design and optimization of broad electrochemical energy devices.

A central quest in hydrogen electrochemical energy devices, such as proton exchange membrane fuel cells and electrolysis cells, is the efficient design of high-performance electrodes, especially catalyst layers (CLs).^1,2^ The CL structures dictate charge and mass transfer, hence influencing performance and durability. The CL design generally involves an optimal interplay between multiscale catalyst nanoparticles, carbon supports and ionomer thin films.^3^ Nevertheless, we are limited by the resolution of advanced imaging tomography and prohibitive electrode synthesis cost to experimentally explore CL design space.^4,5^ Therefore, we need different methods that can cost-effectively scrutinize high-dimensionality design space for optimal CL morphology. Current digital tools for CL design are either low-fidelity or, if high-fidelity, typically are inefficient because of tremendous cost for digital CL nanostructure preparation and performance evaluation.^6−8^ Here, we combine recent advances in generative artificial intelligence and data-centric surrogate modeling techniques to develop a unified generation and optimization framework that can be applied jointly to design multiscale electrode nanostructures beyond CLs.

Reasonable representation of multiscale CL nanostructures and performance evaluation are the foundation for reliable digital CL design and optimization.^9−12^ Regarding structure representation, high-quality representation of multiscale CL nanostructures suffers the “curse of dimensionality” since high resolution is required to resolve the smallest-scale material morphology among platinum particles (around 2–3 nm), carbon supports (around 30–50 nm) and ionomer films (thickness 3–7 nm).^12^ In addition, the region of interest (ROI) needs to be sufficiently large to include relevant spatial interactions among multiple components. For instance, the nanostructures of a 200 nm × 200 nm × 200 nm CL require one hundred million voxels to be resolved at 0.4 nm by electron tomography at cryogenic temperature (cryo-ET).^12^ The high-dimensionality challenge is only mitigated slightly even with the use of current stochastic digital generation algorithms which apply reasonable assumptions to decrease the necessary resolution.^6−8^ High dimensionality also prevents fast performance evaluation due to massive iterations of partial differential equations across complicated nanostructures. Millions of time steps are required to resolve multiphysics behaviors in multiscale multiphysics CL models, even when accelerated by parallel computing.^13,14^ Therefore, it is critical to seek for a method that can accelerate the nanostructure representation and performance evaluation in high-throughput design and optimization of CLs.

There is recent evidence that generative artificial intelligence (AI) has accelerated the design and optimization of various energy materials.^15−18^ Namely, autoregressive models and multimodal transformers accelerated the design of functional organic molecules and metal–organic frameworks by learning the sequence of molecules;^19,20^ Generative adversarial neural networks (GANs) could quickly generate realistic solid oxide fuel cell (SOFC) electrode microstructures and allowed the efficient search of the electrode design space.^21−23^ However, these methods can only handle small and homogeneous nano/microstructures with small ROI (*e.g*., 64^3^ voxels for SOFC anode, the voxel resolution 65 nm)^23^ and will suffer model collapse and tremendous computational demands if applied to design multiscale CLs. Thus, scalability to large ROI that captures multiscale structural behaviors is crucial for generative AI to practically design CLs.

We address this open challenge with GLIDER, a generative AI framework named as ‘Generative Learning to Inform the Design of Electrodes’. GLIDER is **scalable** to accept diverse input nanostructure dimensions, which is achieved by representing high-dimensional CL nanostructures with low-dimensional latent space, underpinned by a vector quantized-variational autoencoder (VQ-VAE).^24^ Hence, generative AI can efficiently learn low-dimensional latent space and generates latent variables which can be transformed back to high-dimensional CL nanostructures. In addition, GLIDER is **tunable** because it provides both high-fidelity and high-efficiency generation options, allowing users to generate CL nanostructures upon their specific demands. Moreover, GLIDER is robust because it seamlessly integrates physical domain knowledge, *i.e*., CL nanostructure connectivity into the performance surrogate model, providing a solid foundation for more accurate and interpretable predictions. Last, GLIDER is **transferable** because it can be easily applied to design and optimization of other electrode micro/nanostructures, such as SOFC anode and fibrous gas diffusion layers (GDLs). In this study, we particularly showcase how GLIDER successfully designs and optimizes Pt–C-ionomer CLs for low-Pt-loading proton exchange membrane fuel cells (PEMFCs).

## GLIDER Overview

**Figure 1:**
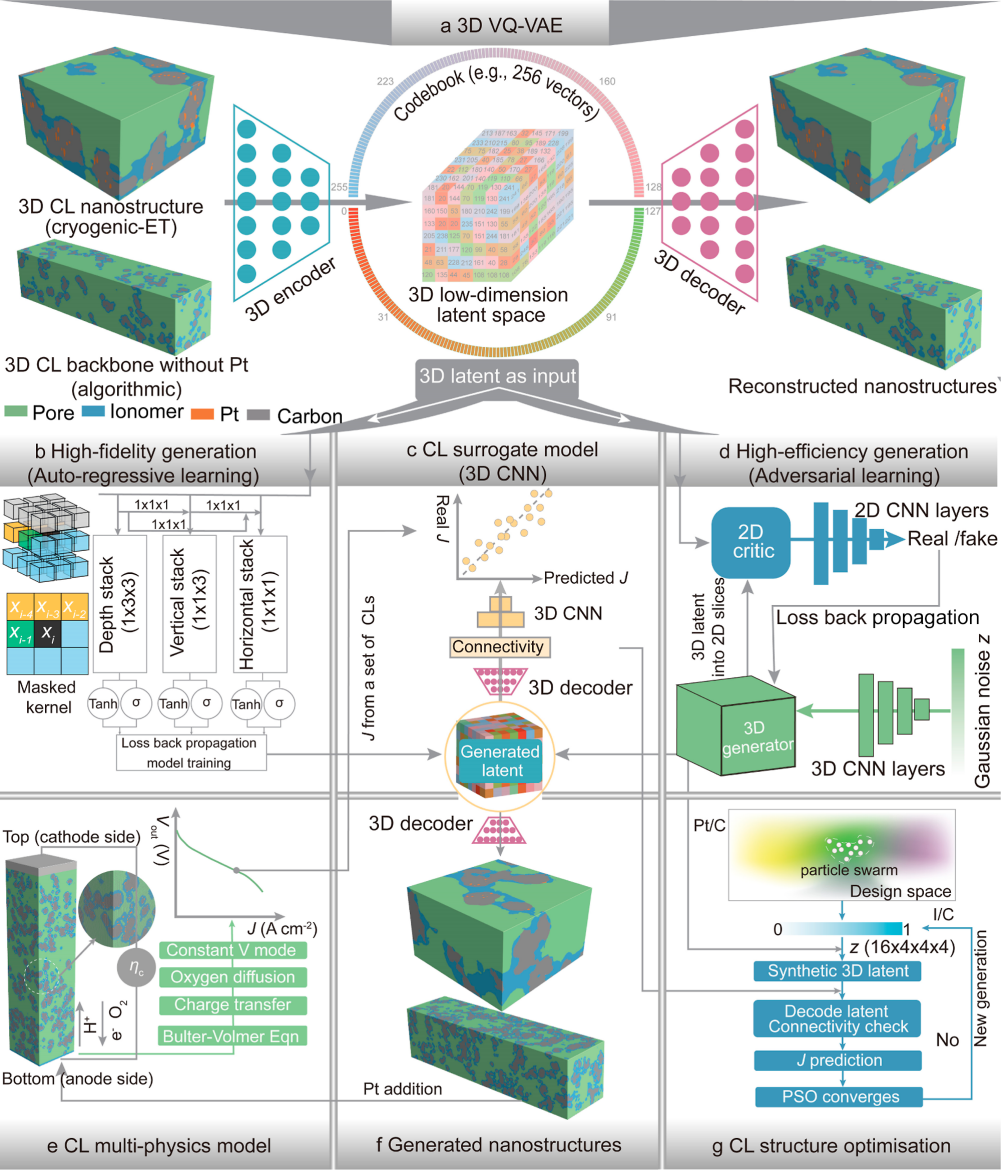
Overview of GLIDER. (a) 3D-VQ-VAE learns to transform high-dimensional CL nanostructures into low-dimensional latent space through the learning of 3D encoder and 3D decoder. Two different kinds of input CL nanostructures are used to train the 3D-VQ-VAE separately for different tasks. (b) An autoregressive generative mode is trained by the 3D latent from (a) and is used to generate 3D latent which are then decoded into high-fidelity 3D CL nanostructures in (f) while the generation is low efficiency. (c) A data-driven CNN model predicts the current density, *J*, of various generated 3D CL nanostructures which are decoded from generated 3D latent. Notably, physical connectivity is embedded in the decoded CLs. (d) A generative adversarial learning model is employed to learn the data structure of the 3D latent from (a) and then is employed to efficiently generate 3D latent which are decoded into high-dimensional 3D CL nanostructures as well. (e) A pore-scale CL model predicts the *J* of a given 3D CL nanostructure by resolving a couple of multiphysics governing equations. The predicted *J* values serve as labels in the training of the data-driven surrogate model in (c). (f) Demonstration of generated 3D CL nanostructures for two different input data sets in (a). (g) Particle swarm optimization for global optimization of pore-scale CL backbone nanostructures with various Pt/C and I/C ratios driven by the *J* from the surrogate model in (c).

GLIDER is a three-stage AI machine which achieves nanostructure generation and optimization by leveraging low-dimensional latent representation space, which consists of six modules in total, as shown in Figure 1. At the first stage, GLIDER reduces the dimensionality of the input CLs with the least information loss. A three-dimensional (3D) vector quantized-variational autoencoder (3D VQ-VAE) transforms multiscale CL nanostructures into a low-dimensional latent space which retains similar meaningful properties of the original input. Accurate transformation in 3D VQ-VAE is enabled by the interactions among a 3D encoder, a 3D decoder and a learnable codebook in between. 3D VQ-VAE can transform CL nanostructures with various types (*e.g*., two CLs with 224 × 384 × 384 and 512 × 128 × 128 voxels in Figure 1a) into low-dimensional 3D latent arrays (*e.g*., 16 × 24 × 24 and 32 × 16 × 16) with the least information loss. The main difference between VAEs and VQ-VAEs is the way they represent latent variables. VAEs employ continuous latent variables, while VQ-VAEs use discrete latent space representation, which is achieved by vector quantization. The latent space representation is constrained to be one of a small set of basis vectors; the set of basis vectors are stored as the “codebook”. At a latent space “location”, the representation is a single index that identifies which basis vector is the most appropriate representation; the representation is therefore quantized in the basis vectors. Thus, the latent space representation becomes a set of indices identifying the codebook’s embedding vectors, and therefore enables a reduction in dimensionality. Both the basis vectors and the indices at each latent space location are learned by the model. The main advantage of VQ-VAEs over VAEs is to learn discrete data like multiphase materials distinguished by discrete voxel values, enabling more stable and interpretable AI techniques.

At the second stage, GLIDER generates 3D CL nanostructures for various demands, *i.e*., high-fidelity or high-efficiency generation, which are generally challenging to simultaneously fulfill. Regarding high-fidelity generation, an autoregressive gated pixel convolutional neural network (Gated PixelCNN)^25^ learns the data structure of the 3D latent from 3D VQ-VAE to generate latent, as shown in Figure 1b. Generated 3D latent is then reconstructed into CL nanostructures by the 3D decoder in Figure 1a, see Figure 1f. High-efficiency generation in GLIDER is achieved by a 3D Wasserstein generative adversarial neural network with gradient penalty (3D WGAN-GP).^20−23^ The adversarial learning between the generator and critic in the 3D WGAN-GP enables the effective learning of the 3D latent generated in the stage 1. The latent from the trained generator is then reconstructed into 3D CL nanostructures by the 3D decoder afterward.

At the last stage, GLIDER efficiently optimizes CL nanostructures by tuning Pt/catalyst (Pt/C) and I/catalyst (I/C) ratios. Here, GLIDER is underpinned by three modules, *i.e*., the high-efficiency CL generator from the stage 2, a data-driven surrogate model for the current density *J* of CLs, as well as a global particle swarm optimization (PSO) algorithm,^26^ see Figure 1c,f for the interaction between the modules. Notably, the training of *J* surrogate model takes the numerical data simulated by a 3D pore-scale CL model in Figure 1e.

## Results and Discussion

### Encoding and Reconstruction of Multiscale CL Nanostructures

**Figure 2:**
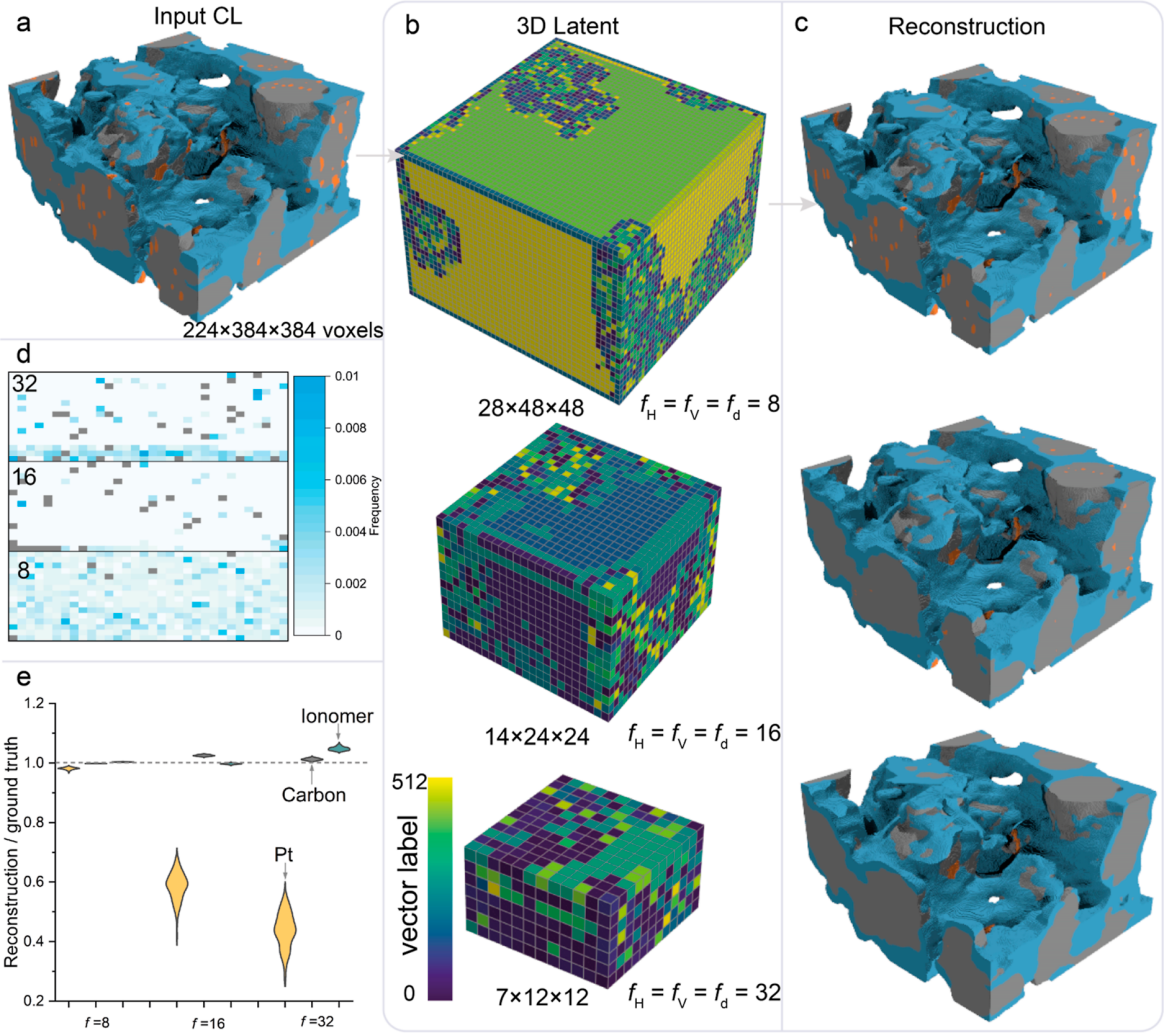
Encoding and decoding CL nanostructures under three compression ratios. (a) A cryogenic-ET CL nanostructure with 224 × 384 × 384 voxels for example (pore phase is hidden to show more inside details). (b) Encoded 3D latent colored by vector labels of the codebook where 512 vectors were employed in total. (c) Reconstructed CL nanostructures by the 3D decoder. (d) Statistical frequency of 512 vector labels used in 100 test CL samples for *f* = 8, 16 and 32, respectively. Here, gray spots indicate frequency is far larger than 0.01, indicating the intensive usage of the specific vector. The 512 vector labels are arranged into a 32 × 16 matrix for the three cases, respectively. (e) The comparison between real and reconstructed volume fraction of ionomer, carbon and Pt.

We initially showcase the capability of GLIDER in effectively compressing real multiscale nanostructures obtained through electron tomography at cryogenic temperatures (cryogenic-ET) into low-dimensional latent representations. We explore the performance of GLIDER under compression ratios of *f* = 8, 16, 32 along each dimension of CLs. Here, CLs are equally compressed along the depth-wise, vertical and horizontal directions. However, compression can be heterogeneous on demand. The real CL nanostructures, characterized by a size of 224 × 384 × 384 voxels as depicted in Figure 2a, are encoded into 3D latent representations with varying sizes corresponding to the three *f* values. A smaller compression ratio, such as *f* = 8, leads to a larger 3D latent size of 28 × 48 × 48, while a larger ratio like *f* = 32 results in a smaller 3D latent size of 7 × 12 × 12, as illustrated in Figure 2b. Notably, under higher compression ratios (*f* = 32), associating latent distribution with real material voxels becomes challenging, in contrast to the clearer identification of large pores in the latent when using lower compression ratios like *f* = 8. Furthermore, our observations reveal distinct patterns in the utilization of vector codebooks, as depicted in Figures 2d and S1a. The usage of the codebook generally declines when the compression ratio *f* increases, especially when *f* increases from very small to large values. This is because many local structural features are preserved after light compression and thus the model needs more types of vectors in the codebook to represent diverse features. However, the usage of the codebook drops under significantly large compression because some minor local features are lost during compression and thus the model requires fewer vectors in the codebook. Specifically, when *f* = 8, nearly all 512 vectors in the codebook are actively involved in reconstructing CLs, showcasing a powerful capacity to represent high-dimensional CLs in a low-dimensional latent space. Because each vector in the codebook can represent a specific feature of the original CLs. Conversely, a significant drop in vector engagement is observed with higher compression ratios (*f* = 16 and *f* = 32), indicating a more selective use of vectors in these cases.

3D latent with various sizes are then reconstructed into high-dimensional multiscale CL nanostructures by the 3D decoder. It is seen from the 3D CL morphology in Figure 2c that GLIDER visually reconstructed low-dimensional latent representations to real CL nanostructures under three *f* values. However, we observe Pt particles were lost somewhere, especially in case *f* = 32. To quantify the reconstruction accuracy, we further compare the volume fraction of ionomer, carbon and Pt against the input, as show in Figure 2e. *f* = 8 shows good performance by reconstructing all components accurately. As *f* increases, though ionomer and carbon phases are reconstructed with reasonable accuracy, nearly half Pt particles disappear in the reconstructed CLs. A series of slices through the CL are shown in Figure S1b to highlight the Pt loss under high *f*. It is seen that disappeared Pt particles tend to have smaller equivalent diameters. The reason for significant Pt loss under high *f* is attributed to the nature of CNNs which are applied in the encoder and decoder to extract data features. Information loss is inevitable after the input passes through multiple convolution layers. The 3D distributions of the reconstructed errors for the CLs under three *f* values are shown in Figure S1c.

We further validate the transferability of GLIDER in encoding and decoding various fuel cell electrode microstructures. Specifically, we apply GLIDER to compress and reconstruct GDLs, characterized by randomly stacked fibers. The reconstructed GDL morphologies under different compression ratios are presented in Figure S2. The results reveal that GLIDER maintains accurate reconstruction with diminishing precision as *f* increases, consistent with observations in CLs. Notably, due to the relatively limited material components in GDLs, such as fibers and pores, the reconstruction process is less challenging compared to CL reconstruction. This is evident in the achieved reconstruction accuracy exceeding 90% for GDLs, as illustrated in Figure S2c. Additionally, GLIDER is demonstrated in reconstructing SOFC anode microstructures, showcasing accurate reconstruction of Nickel (Ni) and Yttria-stabilized zirconia (YSZ) as depicted in Figure S3.

### Generating Cryogenic-ET CL Nanostructures from Low-Dimensional Latent Space

Our initial demonstration focuses on the capacity of GLIDER to generate and high-quality cryo-ET CL nanostructures. The generation process involves passing the index of low-dimensional latent output from the 3D encoder to the high-fidelity generator, which, in this case, is an autoregressive model known as 3D Gated PixelCNN (as illustrated in Figure 1b). Following training, the Gated PixelCNN exhibits the ability to generate realistic 3D CL nanostructures across various compression ratios (*f* = 8, 16, and 32). Notably, these *f* values are consistent across the three dimensions of CLs in all cases of the section.

**Figure 3:**
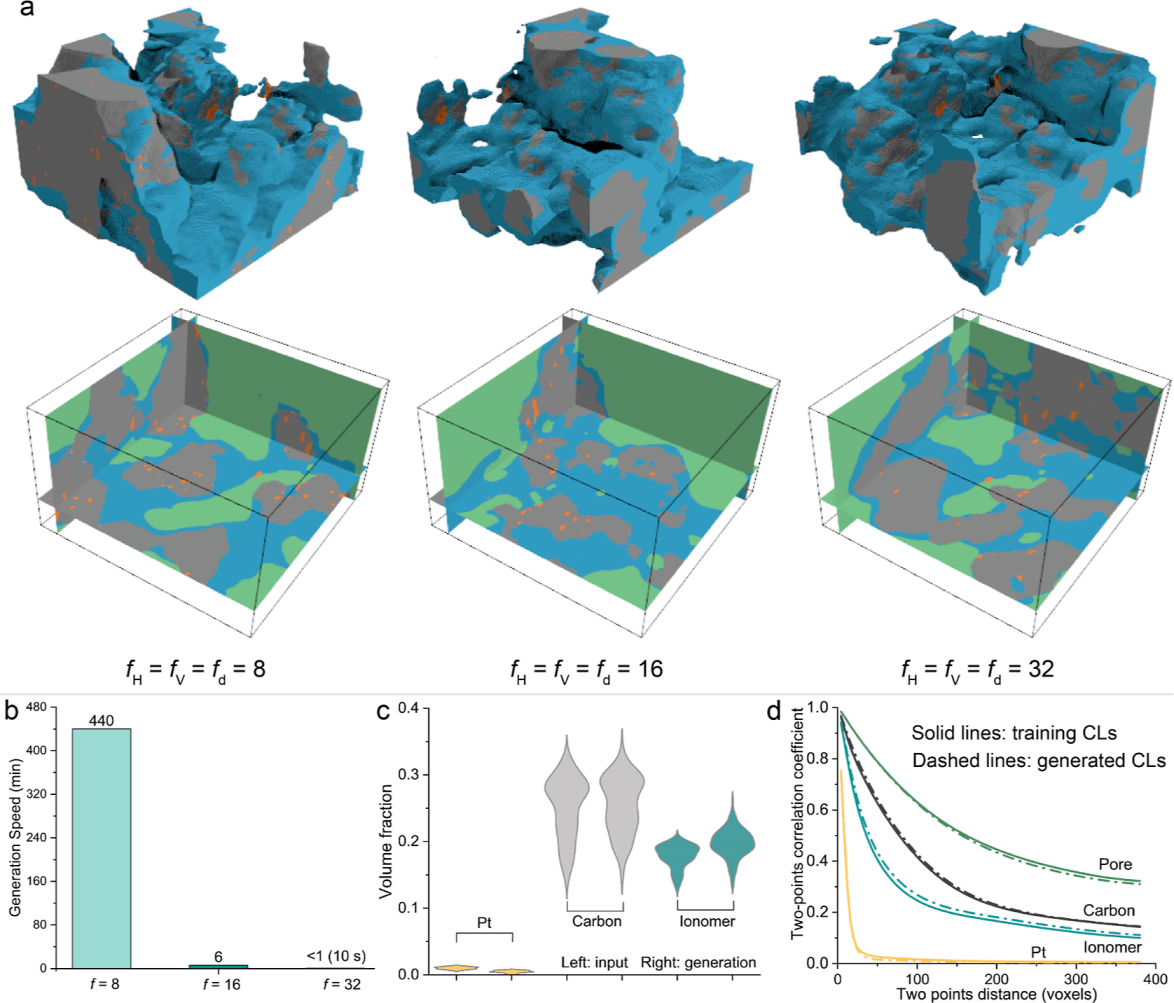
Performance of GLIDER on generating CL nanostructures under high-fidelity generation mode using *f* = 16 in the depth-wise, horizontal and vertical directions. (a) Generated CL nanostructures in cases *f* = 8, 16 and 32, respectively. (b) Generation efficiency of a single CL in cases *f* = 8, 16, and 32 under high-fidelity generation mode. (c) Comparison of generated volume fraction of pore, ionomer, carbon and Pt against real CLs in the case *f* = 16. Statistical analyses are conducted over 100 generated and real CLs, respectively. (d) Two-point correlation coefficient curves of 100 real and 100 generated CLs in the case *f* = 16. The generated curve for Pt phase is mostly overlapped by the training data curve.

The visually aligned comparison between generated CLs and real CLs is evident in Figures 2a and 3a. The synthetic CLs exhibit Pt particles embedded into carbon clusters, predominantly covered by a thin ionomer film, mirroring the characteristics of real CLs depicted in Figure 2a. Moreover, the representation includes a substantial proportion of pores within CL regions, as expected. Critically, the ionomer film demonstrates good connectivity, ensuring the presence of a continuous proton transport path. Due to the inherent loss of Pt particles during the preparation of the low-dimensional latent, observations in CLs generated with higher compression ratios (high *f* values) reveal fewer Pt particles, as illustrated in CL slices in Figure 3a. This emphasizes the trade-off between compression efficiency and the preservation of detailed features in the generated nanostructures.

Next, we focus on the comparison of the generation speed of GLIDER across three different compression ratios (*f* values). Figure 3b illustrates the time taken to generate a single high-fidelity CL for cases where *f* equals 8, 16, and 32. Notably, *f* = 8 requires the most extended duration, approaching nearly 8 h, while *f* = 32 exhibits the swiftest generation time, taking just around 10 s. This considerable variation in generation speed is attributed to the autoregressive nature of the process, where the Gated PixelCNN generates each voxel sequentially, and the generation of each voxel depends on all previously generated voxels. Notably, the generation cost becomes notably significant when dealing with larger input latent dimensions, such as 28 × 48 × 48 in the case of *f* = 8.

To trade off efficiency and accuracy, we conduct a quantitative assessment exclusively for the case of *f* = 16, where the generation efficiency is deemed acceptable. Figure 3c provides a comparison between the volume fractions of Pt, carbon, and ionomer in the generated CLs as opposed to the training CLs. Notably, the volume fraction of carbon remains nearly identical across over 100 generated samples, with slight differences observed in the volume fraction of ionomer. As anticipated, the generated volume fraction of Pt was lower due to Pt loss during the dimensionality reduction in the 3D encoder. To further validate the spatial fidelity of the generated CLs, we investigated the two-point correlation curves for Pt, ionomer, carbon, and pore regions across 100 generated CLs, as shown in Figure 3d. The observed alignment between the generated curves and those of real CLs suggests that the spatial structures of the generated CLs closely resemble those of real CLs.

**Figure 4:**
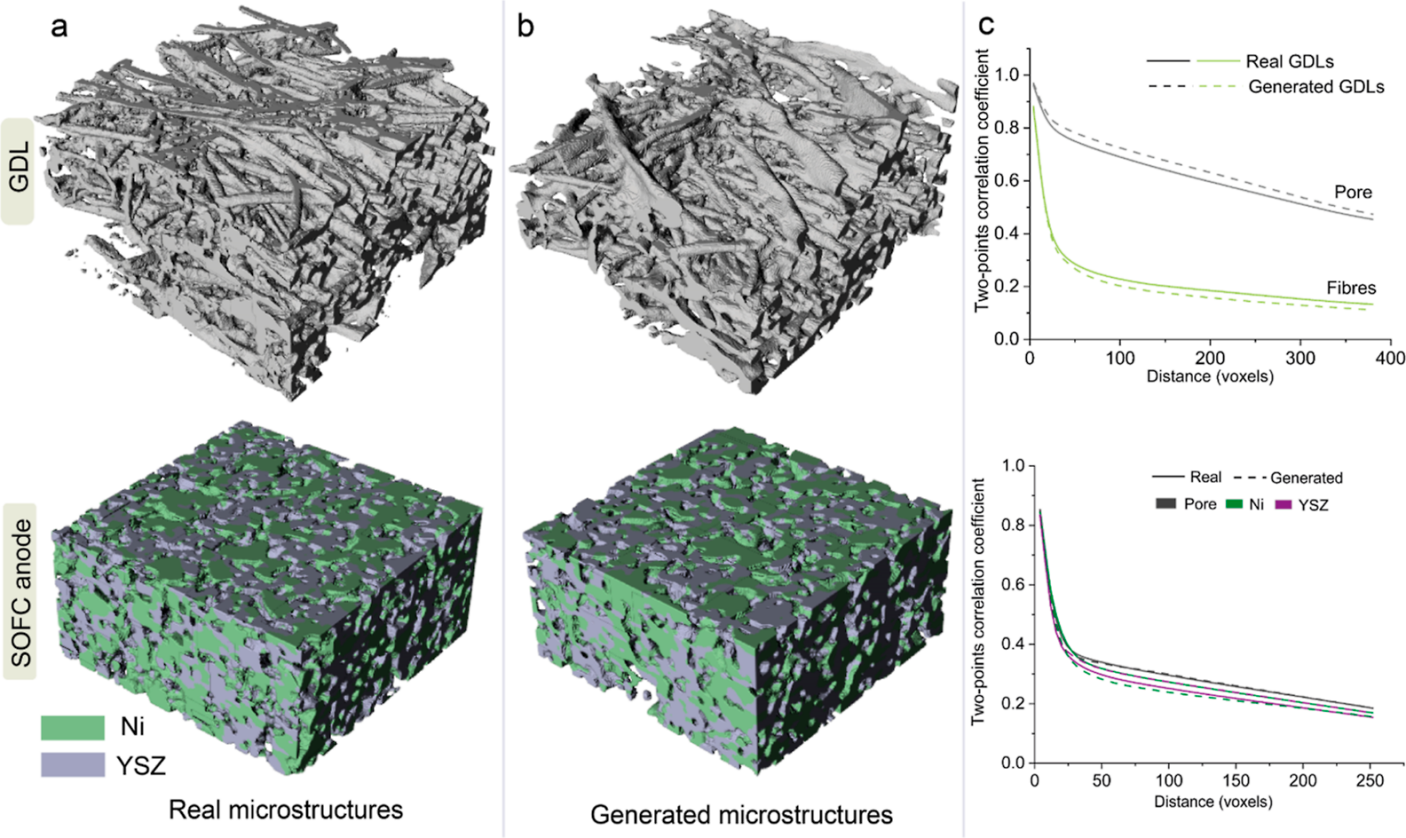
Transferability of GLIDER demonstrated in the generation of GDLs and microporous SOFC anode. (a) Real fibrous GDL and SOFC anode microstructure in the training data set that were experimentally imaged by X-ray nano tomography and FIB-SEM, respectively. The GDL size is 224 × 384 × 384 (resolution 1.33 μm). Regarding SOFC anode, it has a size of 128 × 256 × 256 (resolution 65 nm) (b) Top: a generated GDL in high-fidelity mode under *f* = 16 along three directions. Bottom: a generated SOFC anode in high-fidelity mode under *f*_H_ = 8, *f*_V_ = 8, and *f*_d_ = 4. (c) Two-point correlation curves which are analyzed over 10 generated and training samples. Top: GDL; Bottom: SOFC anode.

The generation capability of GLIDER extends seamlessly to other nanoscale and microscale electrode microstructures with the simple substitution of the training data set. To illustrate this transferability, we showcase the generation of GDLs and SOFC anode microstructures, utilizing realistic GDLs and SOFC electrode microstructures as the training data,^27,28^ see Figure 4. Even when presented with microstructures exhibiting different structural characteristics from CLs, GLIDER consistently exhibits the ability to generate high-quality fibrous GDLs and SOFC anode microstructures. This emphasizes the versatility and adaptability of GLIDER across diverse nanoscale electrode systems. However, unrealistic structural features are occasionally observed in the generated microstructures, *e.g*., the isolated YSZ particle in Figure 4b. This is caused by the isolated YSZ regions existing in the training data sets. Because hundreds of 3D SOFC anode training microstructures are sampled by randomly cutting a super large anode sample. Thus, isolated material regions are possible to appear near the domain edges. The check of the structural connectivity can detect the microstructures without structural supports and thus can potentially enhance physical realism of generated microstructures. In addition, reducing unrealistic structural features in the training data sets is helpful to improve the generated quality.

The high-quality electrode and CL nanostructures generated by GLIDER offer significant value for educational purposes in fuel cell manufacturing. GLIDER facilitates the exploration of intricate interactions among multiple key material components of CLs through virtual reality,^29,30^ providing users with a diverse range of nanostructures. While the powerful generation ability of GLIDER has been established, it is important to note that the diversity in generation depends on the input diversity of CL nanostructures. Unfortunately, achieving such diversity requires access to specific experimental facilities for imaging various CL samples, which is both cost-prohibitive and time-consuming. Typically, diverse CL generation involves the implementation of logic-based algorithms, which incrementally introduce multiple material components into a CL domain until the target fraction is satisfied. Additionally, despite employing an intermediate compression ratio *f*, the high-fidelity generation speed of GLIDER remains relatively slow. This limitation hinders the efficiency of exploring a large design space. Consequently, the application of GLIDER for the digital design and optimization of cryo-ET CL nanostructures presents a challenging endeavor. Addressing these challenges would be crucial for leveraging the full potential of GLIDER in advancing the digital design and optimization of complex CL nanostructures efficiently.

### Generating Algorithmic CL Nanostructures from Low-Dimensional Latent Space

**Figure 5:**
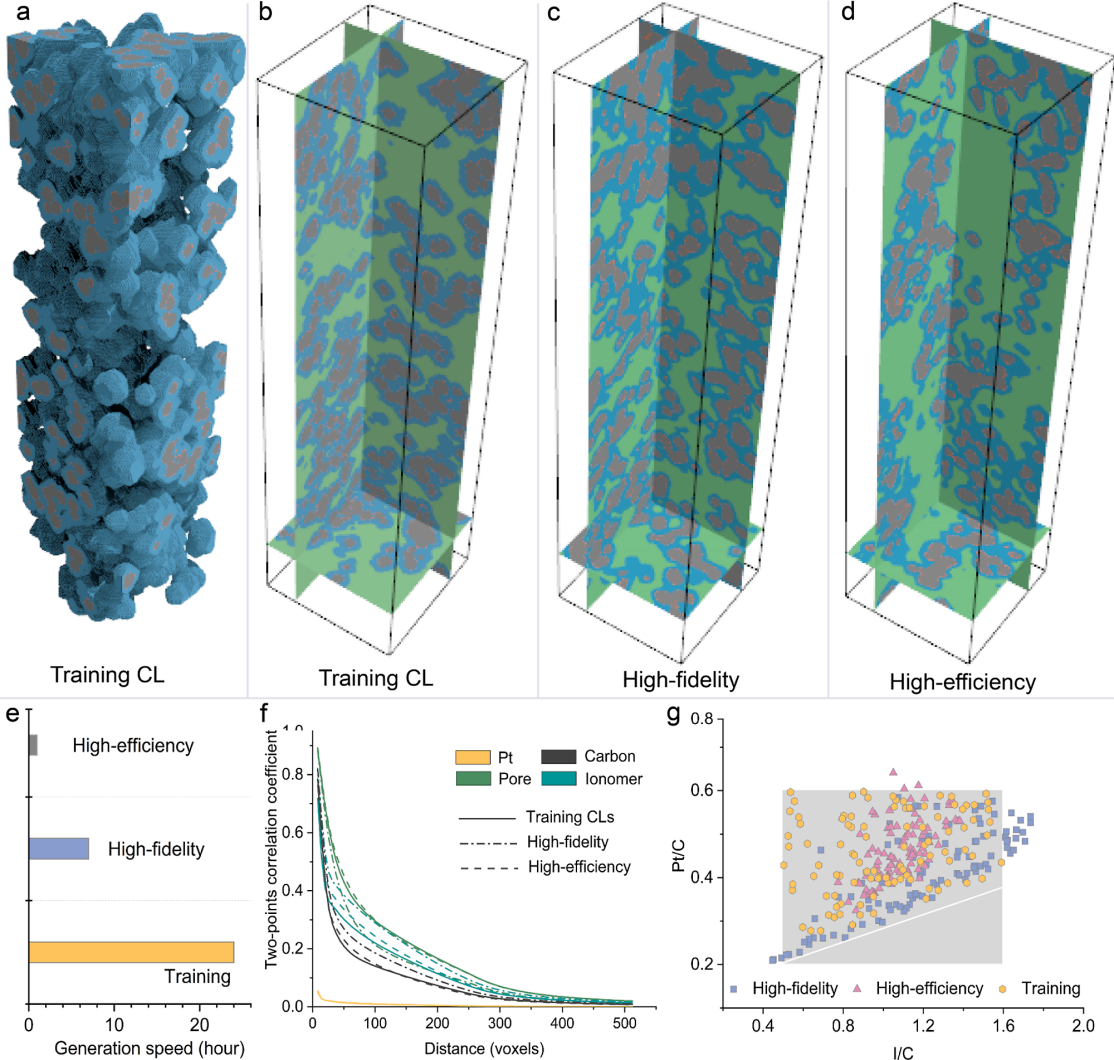
Performance of GLIDER for logic CL generation (512 × 128 × 128 voxels, resolution 3.5 nm). (a,b) The morphology of a CL generated by a conventional logic CL generation algorithm. (c) Generated CL under high-fidelity mode (Gated PixelCNN). (d) Generated CL under high-efficiency mode (WGAN-GP). (e) Generation cost under high-efficiency high-fidelity modes and using a conventional logic generation algorithm. (f) Two-point correlation curves of generated CLs under high-fidelity and high-efficiency modes, as well as from a conventional logic CL generation algorithm. (g) The comparison of generated Pt/C and I/C ratios under high-efficiency and high-fidelity modes against training CLs, where the compression ratios along three dimensions are *f*_d_ = 32, *f*_h_ = 8 and *f*_v_ = 8. The gray box indicates the area with the Pt/C ratio ranging from 0.2 to 0.6 and the I/C ratio ranging from 0.5 to 1.6. Notably, the gray box represents the upper and lower Pt/C and I/C ratios of the training CL nanostructures obtained from the stochastic algorithm.

To further apply GLIDER to generate algorithmic CLs, GLIDER incorporates another generation mode, *i.e*., high-efficiency generative adversarial learning, to accelerate the generation of CL nanostructures. In this approach, we specifically targeted CL nanostructures from logic generation algorithms, an example of which is shown in Figure 5a,b. Notably, logic CL generation algorithms often assume uniform diameters for Pt particles and discretize a Pt particle by using a voxel. However, this assumption limits the GLIDER to directly generate CLs with Pt particles fully resolved, as substantial Pt loss during encoding and decoding processes was observed as illustrated in Figure S4.

To efficiently generate diverse CLs with Pt particles, GLIDER employs a two-stage method for logic CL nanostructure generation. In the first stage, GLIDER generates the backbones of logic CLs without Pt particles. This involves transforming the original CL backbones with the size 512 × 128 × 128 voxels (resolution 3.5 nm) into a low-dimensional latent space of 16 × 16 × 16, utilizing heterogeneous compression ratios along three directions (*f*_d_ = 32 and *f*_h_ = *f*_v_ = 8). The generative adversarial learning, specifically using WGAN-GP, then learns from the low-dimensional latent space to generate latent representations, subsequently decoded into 3D CL backbones. In the second stage, GLIDER randomly adds Pt particles to the CL backbone, similar to the same assumptions as conventional logic CL generation algorithms. Figure 5c,d showcase the logic CLs generated by GLIDER, with dimensions large enough to allow for the observation of reasonable spatial distribution of carbon, ionomer, and Pt. This innovative two-stage approach effectively combines the strengths of generative AI and logic generation algorithms to enhance the accuracy and efficiency of CL nanostructure generation.

GLIDER significantly leaps forward in the efficiency of logic CL nanostructure generation. GLIDER only took 1 h to generate 100 CLs, a task that would take the conventional logic algorithm nearly 24 h, see Figure 5e. Beyond high generation efficiency, GLIDER ensures the quality of the produced CLs matches that of the training CLs. This is demonstrated by the correlation curves among Pt, carbon, ionomer, and pore regions across 100 samples, as depicted in Figure 5f. To further investigate generation ability of GLIDER, we conduct a comparison under high-fidelity and high-efficiency modes. These modes leverage Gated PixelCNN and WGAN-GP to learn low-dimensional latent representations, respectively. Notably, even under high-fidelity mode, GLIDER maintains an efficient generation pace, requiring only 7 h for 100 CLs. Although slightly deviating from the training curves due to varying Pt/C and I/C ratios in the generated CL distributions, this mode still outperforms conventional high-fidelity methods in terms of time efficiency. Figure 5g offers a comprehensive analysis of the generated CLs under both modes against training CLs, focusing on Pt/C and I/C ratios across 100 samples. Notably, training CLs span a region where Pt/C ranges from 0.2 to 0.6 and I/C ranges from 0.5 to 1.6. In contrast, high-efficiency mode concentrates CLs in the middle of this spectrum, while high-fidelity mode produces more diverse CLs, spanning a wider range of Pt/C and I/C ratios, albeit with a notable absence in the top-left corner of the distribution, as illustrated in Figure 5g.

We extend our exploration to assess the ability of GLIDER to generate logic CLs from latent with large dimension, specifically employing a latent size 32^3^. The rationale behind utilizing larger latent dimensions is the potential reduction of information loss during the encoding and decoding processes. To begin this investigation, we initiate a comparison of the generation performance of GLIDER under the high-efficiency mode. Figure S5a depicts the morphological characteristics of generated logic CLs, decoded from a larger latent size 32^3^. Despite capturing the interplay among the four material components, the visual quality falls short when compared to CLs generated from a smaller latent size 16^3^, as illustrated in Figure 5d. Notably, the ionomer film exhibits a wider spread in the case of the latent size 32^3^, a deviation from the expected and established ionomer morphologies in logically generated CLs. This visual inconsistency is further substantiated through two-point correlation analysis and an examination of the distributions of generated Pt/C and I/C ratios. The reason for GLIDER not being able to generate high-quality CLs from large latent size is because WGAN-GP is limited to capturing spatial correlations among long-distance latent vectors, which is the strength of autoregressive learning in high-fidelity mode on the contrary. This is confirmed by employing GLIDER to generate high-quality logic CLs by using a latent size of 32^3^ under high fidelity, as shown in Figure S5b. However, the computational efficiency (3 h for a CL) is still inferior to traditional logic algorithms. This limitation can possibly be lifted by optimizing the inference processes of Gated PixelCNN in the future.

### Optimizing Low Pt Loading CL Nanostructures

Reducing the Pt loading without sacrificing performance is highly desirable to meet the long-term sustainable target of 5 g_Pt_ per vehicle.^31^ To reduce the oxygen-related mass transport resistance which is deemed to be one main attribute of performance scarification, identifying the optimal Pt/C and I/C ratios are crucial for manufacturing CLs with low Pt loadings. However, previous studies mainly employed macroscopic models as optimization tools, which are limited to reflect nanoscale interplay between ionomer film and carbon frameworks.^32,33^ Here, we demonstrate that GLIDER carried out comprehensive exploration of design parameters of Pt/C and I/C ratios for the CL with Pt loading of 0.05 mg cm^–2^ by integrating their CL performance surrogate model and a PSO algorithm.

**Figure 6:**
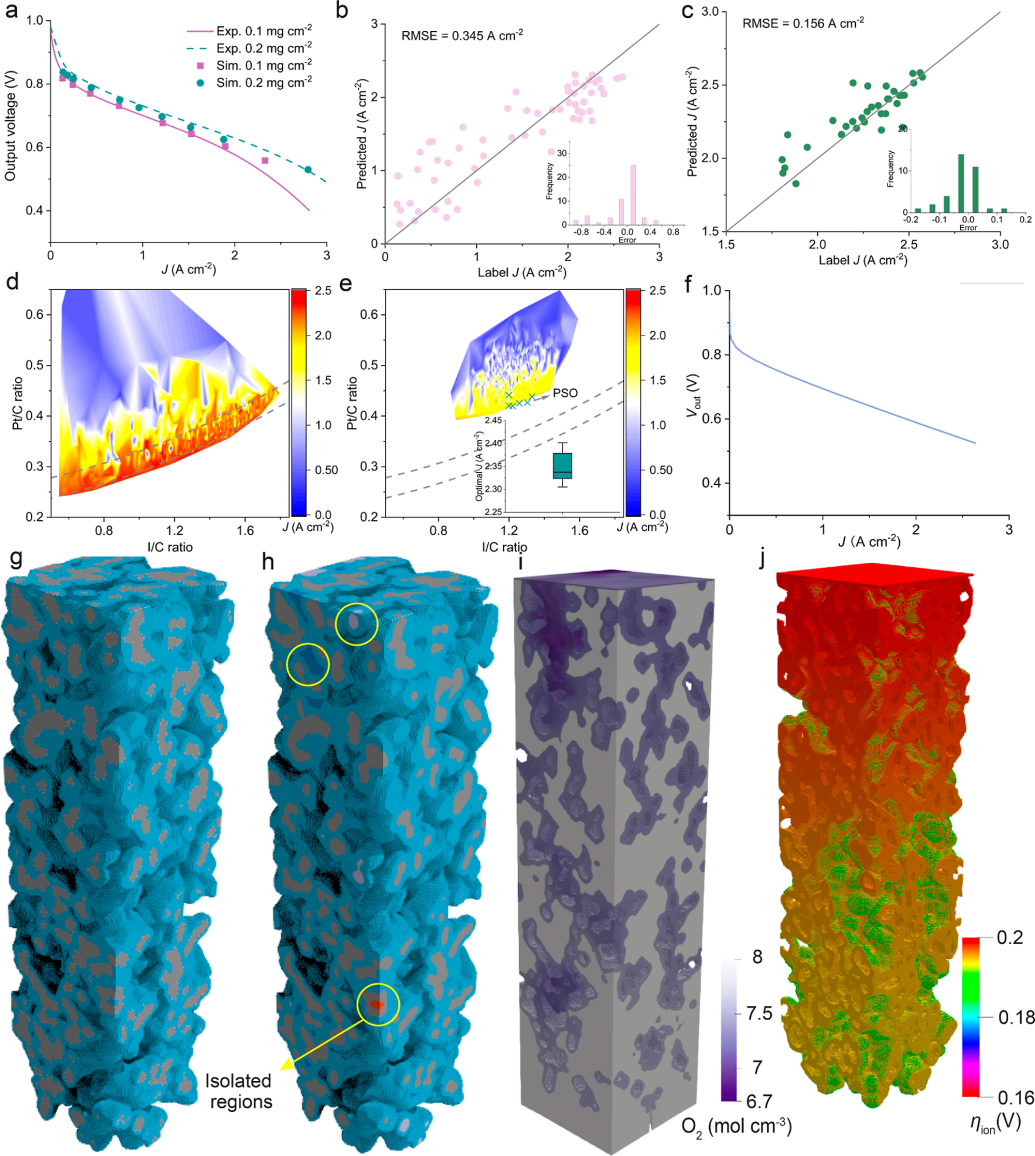
GLIDER optimization of CL morphology based on Pt/C and I/C ratios. (a) Experimental validation of the pore-scale multiphysics CL model under two Pt loadings. (b,c) The accuracy and error distribution of the *J* surrogate model of the CL based on two training data sets: (i) training data set with *J* > 0.1 A cm^–2^ and (ii) training data set with *J* > 1.5 A cm^–2^. (d,e) The contour of *J* mapped (η_cathode_ = 0.2 V) by the surrogated model under high-fidelity and high-efficiency generation modes, respectively. The crosses in Figure (e) refer to the optimal Pt/C and I/C ratios identified by the PSO algorithm. (f) Optimized *J*–*V* curve screened by GLIDER under high-fidelity generation. (g) Morphology of the optimal CL screened by GLIDER under high-fidelity generation. (h) Connected and isolated components in (g). (i,j) Contour of oxygen distribution of the optimal CL operating under η_cathode_ = 0.2 V and the corresponding *J* = 2.64 A cm^–2^.

The performance of a nano porous CL is governed by a series of partial differential equations which generally take high computational cost to resolve. To enable the fast prediction of the CL performance, *i.e*., current density *J* under a given voltage. GLIDER predicts the performance of generated CLs quickly through a data-driven surrogate model of *J* underpinned by a pore-scale multiphysics CL model and a 3D CNN which takes decoded CL nanostructures as input. The pore-scale multiphysics CL model is validated against experimental polarization curves^34^ under the Pt loading 0.1 and 0.2 mg cm^–2^, respectively, see Figure 6a. The pore-scale CL model aligns with the experimental curves well under the two Pt loadings, ensuring the reliability of the training data collected from the model.

We then train the 3D CNN with a range of CL nanostructures labeled by their *J*. We found that embedding the physical feature—connectivity of CL material components into training CLs is crucial to improve the accuracy of the surrogate model. Connectivity is embedded by highlighting connected components with different voxel values, as shown in Figure 6h. We employ two strategies to train the surrogate model to trade off between the model accuracy and generalization due to the wide range of complex training CL microstructures.^23^ The first strategy that is employed takes broad training CLs with *J* > 0.1 A cm^–2^. The accuracy of the surrogate model predicting the test data set and the training data set are shown in Figures 6b and S6, respectively. We observe that the surrogate model displays acceptable accuracy under high *J* (*J* > 1.5 A cm^–2^) but deviates from the real under low *J* (*J* < 1 A cm^–2^). The majority of predicted J show errors within ±10% and big errors (>20%) happen to low *J* range. This is resulted from the limited training data set (less than 200 CLs) which is significantly imbalanced across the whole *J* range, as well as the significant different characters between high-performance and poor CLs. Meanwhile, poor CLs could play as noisy samples and impact the model prediction on high-performance CLs. In the second strategy, we further train the surrogate with training samples of a narrow *J* range which is larger than 1.5 A cm^–2^. As shown in Figure 6c, the surrogate model outperforms itself trained on the broader *J* data set, showing much lower error than ±5% for more than 70% test CLs. In both strategies, the trained surrogate model in GLIDER can quickly predict the *J* of a generated CL less than 1 s, enabling an efficient screening of broad CL design space. Though the whole *J* training strategy does not show good accuracy for low *J* range, which is irrelevant for the CL global optimization, the diversity of the data set could enhance the model generalization.

We evaluate the optimization performance of GLIDER under two generation modes. Figure 6d shows the *J* map of 1,000 CLs generated under high-fidelity mode. The map indicates that the high-performance CLs are attributed to good alignment between Pt/C and I/C ratios. The *J* map reasonably correlates Pt/C and I/C ratios because excessive ionomer (high I/C ratio) and low fraction of carbon supports (low Pt/C ratio) result in high mass and charge transport resistance. To quantify the correlation between Pt/C and I/C ratios for high-performance CLs, we fit CLs with *J* > 2.4 A cm^–2^. The optimal area is depicted by two parallel functions as below
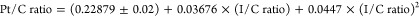
1

Furthermore, we identify the optimal CL from the top 10 which are analyzed by the pore-scale model again as shown in Figure 6f,g. However, the optimization efficiency of GLIDER under high-fidelity mode was low, which took around 70 h to assess 1,000 generated CLs. In contrast, GLIDER performs fast under high-efficiency mode. GLIDER only took 20 min to sample 1,000 CLs under high-efficiency mode, as shown in Figure 6e. The *J* map shows that both high Pt/C and I/C ratios deteriorate CL performance significantly, which is overlooked by high-fidelity optimization in Figure 6d. Whereas, high *J* area predicted by GLIDER under high-efficiency mode is away from the fitted curve. The different *J* maps under different generation modes are caused by the different sampling space learned by the Gated PixelCNN and generative adversarial neural network, which was previously compared in Figure 5g. Notably, the concentration loss under high operating current density is slight in the polarization curves of the modeled CLs, indicated by the linear drop of the output voltage against *J*. This is because the top boundary of the computational domain, as shown in Figure 1e, was set as fixed oxygen concentration and thus can supply efficient oxygen for the electrochemical reactions inside CLs.

Another advantage of GLIDER optimizing CLs under high-efficiency mode is that it could intelligently search the optimal instead of looping every possible CLs which are commonly done by brute-force exploration. PSO successfully identifies several optimal CLs which locate around the high *J* area, as seen in Figure 6e. Though GLIDER shows strengths and limits of optimization under two generation modes, they complement each other. A comprehensive understanding of the correlation between various Pt/C and I/C pairs and *J* can be achieved by the joint application of the two modes.

Fast and accurate multiscale CL design is among the most desired needs of developing low Pt loading and durable fuel cell technologies. Here we have demonstrated this need can be addressed by GLIDER that seamlessly integrates multiple CL generators and a performance surrogate model to efficiently generate and optimize CL nanostructures from the perspective of 3D morphology.

GLIDER achieves the generation of real CL nanostructures by applying high-fidelity autoregressive learning. GLIDER was also transferred to generate other relevant electrode microstructures in fuel cells, such as GDLs and SOFC anode. Finally, GLIDER further accelerated the logic CL generation through two generation modes and optimized the relevant manufacturing parameters Pt/C and I/C ratios. We believe that these results could soon have practical benefits to low-Pt-loading fuel cell design and optimization.

GLIDER is enabled by several key innovations: advances in CL nanostructure generation, transforming high-dimensional CLs into low-dimensional latent representations; providing high-fidelity and high-efficiency options for various CL generation demands; robust prediction of CL performance, enabled by a physics-embedded data-driven surrogate CNN; efficient optimization of manufacturing parameters for low-Pt-loading CLs, and providing both optimal parameters as well as the correlated map among Pt/C, I/C and *J*.

A limitation of GLIDER is that it cannot reserve the super small features, *e.g*., Pt particles resolved by one voxel, when compressing high-dimensional CLs under extremely high compression ratios. Though this limitation was addressed in the study by a hybrid CL generation approach, an important future step is to address the issue through hierarchical VQ-VAE architecture which learns large- and small-scale features individually.

## Conclusions

We have introduced GLIDER, a generative AI machine that is readily applied to generate and design multiscale catalyst layers (CLs). GLIDER provides multiple generation capabilities to meet various demands for nanostructure generation. The efficient generation of GLIDER further enabled the efficient optimization of two CL design parameters, Pt/C and I/C ratios, underpinned by a surrogate performance model which is driven by a multiphysics, multiscale CL model. More broadly, GLIDER is an AI machine that can be transferred to various micro/nano porous electrode structures. We have shown this transferability by using fibrous gas diffusion layers and solid oxide fuel cell anode. GLIDER also keeps evolutionary by reserving the interface to connect more powerful generative AI emerging in the future. This deep learning model can be a promising efficient tool to accelerate the research and development beyond fuel cell CLs.

Owing to the rapid development of generative AI, the application of hierarchical VQ-VAEs could possibly address the challenges of high-fidelity multiscale nanostructure generation, *e.g*., VQ-VAE-2^35^ and latent diffusion models.^36^ These models adopt a multiscale autoencoder to capture global and local features and could encode structures into two hierarchical latent spaces. Then, the high-fidelity nanostructures can finally be generated by the global latent representation conditioned on local latent representation. However, their computational costs and performance have not been fully investigated yet and could be a future direction for the multiscale generation of nanostructures.

## Methods

### 3D VQ-VAE

3D VQ-VAE is a variant of variational autoencoder that transforms high-dimensional 3D nanostructures into a low-dimensional and discrete latent space by using vector quantization. The codebook is denoted as , where *K* is the number of discrete code vectors and *D* is the dimension of the code vectors. *K* and *D* are set as 512 and 64, respectively. The 3D encoder and decoder consist of a series of 3D convolutional layers and transposed convolutional layers, respectively. The number of convolutional layers depends on the compression ratio *f*. *f* is defined as the ratio of the original dimensional size and the latent size along each dimension after compression. The detailed architecture of a 3D VQ-VAE is shown in Figure S7. During the model training, an original high-dimensional 3D nano CL, denoted as *x*, is first encoded into *z*_e_(*x*) by the 3D encoder. *z*_e_(*x*) is further discretized into *z*_q_(*x*) by looking up the codebook. Afterward, *z*_e_(*x*) passes through the 3D decoder and is decoded into the high-dimensional nano CL. The model parameters of the 3D encoder and decoder are updated by the backpropagation of the following loss
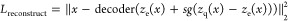
2where *sg* represents the operation of stop gradient. *L*_reconstruct_ measures the error between the input and reconstructed 3D nano CLs. Apart from updating the 3D encoder and decoder, the codebook is updated simultaneously according to the codebook loss
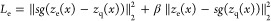
3where β is a hyperparameter, which is 0.25 in the study.

1,100 3D cryogenic CL nanostructures were sampled from a large cryogenic-ET CL data set.^12^ Each sample has a size of 224 × 384 × 384 voxels (resolution 0.4 nm). The split ratio for training and validation data sets is 10:1. When training the 3D VQ-VAE with 3D algorithmic CL backbones, 600 3D CL backbones (without Pt particles) were synthesized by a conventional pore-scale CL synthesis algorithm as described in Supporting Information Note S1. The split ratio for training and validation data sets is 5:1. Each sample has a size of 512 × 128 × 128 voxels (resolution 3.5 nm). Notably, the number of convolutional layers were adjusted accordingly for different demands of compression along three dimensions. The whole data set was split according to the ratio 5:1 for the training and validation. One hot encoding is applied to enable the voxel value types align with the training data. An Adam optimizer with default settings was employed to optimize model parameters. The batch size and learning rate were set as 8 and 3 × 10^–4^, respectively. The 3D VQ-VAE was trained for 400 epochs before applied to subsequent latent generation. The training of VQ-VAE for two types of CLs were implemented on an A100 GPU and each case takes around 24 h.

### Autoregressive Learning

3D Gated PixelCNN is a typical autoregressive generative model which learns the joint distribution of the vector labels of the 3D latent from the encoder in 3D VQ-VAE. The reason of applying PixelCNN here is that PixelCNN performs well in capturing long-range dependencies and correlations among elements in latent space. The joint distribution *p*(*e*) of vector labels over the 3D latent space ***e***^*m*×*n*×*k*^ is expressed as follows
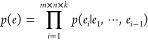
4where *e* represents the input 3D latent. *e*_*i*_ is an arbitrary vector label in the input. *m*, *n* and *k* are the dimensions of ***e***, which is decided by the size of 3D input nanostructures and scaling factor *f*. The label of *e*_*i*_ varies between 0 and the size of codebook *N*, *e.g*., 512 in the study. Then, the Gated PixelCNN was developed to estimate the probability of a given position of the 3D latent space in *N* classes. A masked 3 × 3 × 3 convolutional kernel is designed to enable causal convolution, which means the masked kernel cannot access the vector information below, to the back and the right of a given vector position, as depicted in Figure 1b. The masked convolutional kernel consists of three kernels with different masks, leading to three different kernel sizes of 1 × 3 × 3 in the depth-wise direction, 1 × 1 × 3 in the vertical direction and 1 × 1 × 1 in the horizontal direction. The output of masked convolutional layers then flows through a gated unit which consists of tanh and sigma activation functions, as shown in Figure 1b. The multiple active units in the gated unit allow the model to create highly complex representations of the input.

The loss function of Cross-Entropy is chosen for gradient propagation. An Adam optimizer with default settings was employed for model parameters optimization. The batch size and learning rate are set as 32 and 1 × 10^–3^, respectively. The Gated PixelCNN was trained for 400 epochs in total. Since the computational cost is proportional to O (*m* × *n* × *k*), the Gated PixelCNN was trained on an A100 GPU for the case *f* = 8 (latent size 28 × 48 × 48) and a RTX 3090 for the case of *f* = 16 and 32 (14 × 24 × 24 and 7 × 12 × 12, respectively). The trained model is then able to generate 3D latent by sampling every vector position in sequence. 3D CL nanostructures are eventually obtained by decoding the generated latent. Notably, only the pretrained decoder in Figure 1a is engaged with the Gated PixelCNN.

### Adversarial Learning

3D WAN-GP is a popular generative adversarial learning model. It consists of a 3D *generator* and a 2D *critic*. The 3D generator network maps a 16 × 4 × 4 × 4 Gaussian noise latent into the input space. The 2D *critic* receives either a generated 3D sample or a true 3D nanostructure and minimizes the difference of generated samples with respect to real samples. Here, the generator is trained to fool the *critic*. Notably, to accurately learn the spatial distributions of latent along three dimensions, fake 3D samples were cut into a series of 2D slices along three dimensions, which were subsequentially discriminated by three 2D critics, respectively. To model the above adversarial behaviors, the *generator* and the *critic* networks are trained by applying the following two loss functions respectively
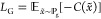
5
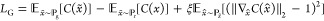
6where *x* is the real data space, which indicates 3D nanostructures for training. *x̃* = *G*(*z*) defines the generated 3D samples from the *generator*, where *z* is the Gaussian noise latent space. *C* denotes the *critic*.  and  denote the generated and real (training) data distributions, respectively. To stabilize the training of WGAN-GP, a gradient penalty is introduced in the model, expressed as the second term in eq 5. Here,  is a random sample with identical dimensional size with *x̃̃*. A hyperparameter ξ = 10 is applied to tune the contribution of the gradient penalty term to the model training. The training data for the 3D WGAN-GP is prepared by the trained 3D encoder in the 3D VQ-VAE, where the original high-dimensional 3D CL nanostructures are encoded into low-dimensional 3D latent space (32 × 32 × 32). The batch size is 8 and 256 for the 3D generator and the 2D critic, respectively. The model is trained for 100 epochs with Adam optimizer. The learning rate, β_1_ and β_2_, were set to 1 × 10^–4^, 0.5 and 0.9, respectively. Detailed model architecture is shown in Figure S8.

### Pore-Scale CL Model

In the following, the governing equations and correlations accounted in the study is presented. We summaries the governing equations for electrochemical reaction kinetics, oxygen diffusion and charge transfer considered in the pore-scale CL model, as well as the boundary conditions and numerical implementation. Model parameters and constants associated with the model are listed in Supporting Information Table S1.

Electrochemical reaction kinetics: The electrochemical reaction rate of oxygen reduction (ORR) in the cathode CL is approximated by the Bulter–Volmer (BV) equation^37^
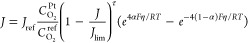
7where *J*_ref_ (A m^–2^) is the ORR exchange current density,  (mol m^–3^) the molar concentration of oxygen around Pt surface,  (mol m^–3^) the reference molar concentration of oxygen, α the charge transfer coefficient, *R* (J K^–1^ mol^–1^) the ideal gas constant, *F* (C mol^–1^) Faraday constant, and *T* (K) the local temperature. As the model is assumed isothermal, a constant *T* = 353.15 K is applied in all cases. η (V) is local overpotential, calculated as follows
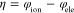
8where φ_ion_ (V) and φ_ele_ (V) are the ionic and electronic potentials, respectively. A correction term  is introduced in eq 7 to mimic limiting current behaviors which enables the model to account for the effects of concentration overpotential under high operating current density.^31^ Here, *J* (A cm^–2^) is the operating current density of the CL. The limiting current density *J*_lim_ (A cm^–2^) is calculated as below
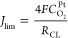
9where *R*_CL_ (s m^–1^) is the mass transport resistance at the interface between Pt surface and ionomer.

Oxygen diffusion: To accurately predict the oxygen diffusion in nano porous CLs, it is imperative to take into account Knudsen diffusion. The local oxygen diffusivity *D*_p_ (m^2^ s^–1^), including Knudsen diffusion, is calculated as follow^38^
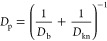
10
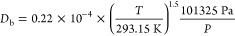
11
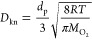
12where *D*_b_ (m^2^ s^–1^) and *D*_kn_ (m^2^ s^–1^) are the bulk and Knudsen oxygen diffusivities, respectively, *P* (Pa) the operating pressure, *d*_p_ (m) the diameter of local pore, π the mathematical constant, and *M*_O_2__ (kg mol^–1^) the molar mass of oxygen. As oxygen dissolves variously in air and ionomer, the concentration drop at the air-ionomer interface is described by Henry’s law, which correlates the oxygen concentration  (mol m^–3^) in pores and  (mol m^–3^) in ionomer via Henry’s coefficient *He* as follows^6^
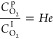
13
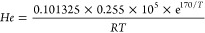
14

To unify the oxygen transport in both pores and ionomer, a scalar *C*_e_ (mol m^–3^) is introduced as the global effective oxygen concentration as below^39−41^
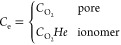
15
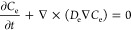
16where *D*_e_ (m^2^ s^–1^) is the effective diffusion coefficient calculated as follows
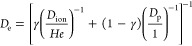
17where γ is an indicator which is only applied to diffusion regions (0 in pores and 1 in ionomer, respectively). *D*_ion_ (m^2^ s^–1^) is the oxygen diffusivity in ionomer, calculated by^6,42^
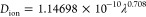
18

It is seen from eq 18 that *D*_ion_ depends on the water content λ, which is assumed constant in the study.

Charge transfer: The transport of proton and electron in the CL is governed by the following two equations
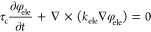
19
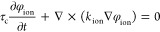
20where *k*_ele_ (S m^–1^) and *k*_ion_ (S m^–1^) are the electronic and ionic conductivities in carbon and ionomer, respectively. It is noted that in eqs 19 and 20, the constant τ_c_ is introduced in the unsteady term to stabilize numerical iterations through the pseudotransient method. The choice of τ_c_ has no effect on the steady solution of the equation. Since water content λ significantly impacts ionic conduction, *k*_ion_ is correlated with λ by the following^43^
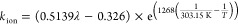
21

Boundary conditions: In terms of oxygen transport, the top of the buffer (see Figure 1e) is set as Dirichlet boundary condition where *C*_e_ = 10.93 mol m^–3^. The interface between ionomer and carbon is set as the fixed flux *J*_O_2__ (mol m^–2^), calculated by
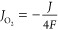
22

Regarding charge transfer, a cathode overpotential η_c_ (V) is applied across the CL (see Figure 1e) by setting φ_ion,bottom_ = η_c_ and φ_ele,top_ = 0. The consumption of proton and electron is considered by applying the fixed flux *J*_ion_ (A m^–2^) and *J*_ele_ (A m^–2^) at the Pt-ionomer and Pt-carbon interfaces, respectively. *J*_ion_ and *J*_ele_ are correlated with *J*_ORR_ as follows
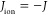
23
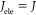
24

Notably, the cell output voltage *V*_out_ (V) is calculated as follows
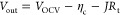
25where *V*_OCV_ (V) is the open circuit voltage of the cell, and *R*_t_ (Ω m^2^) the total charge transfer resistance of the cell except the cathode CL.

Numerical implementation: The computational domain of CLs was prepared by a logic CL generation algorithm, as described in Supporting Information Note S1. The pore-scale model was implemented in an open-source platform OpenFOAM. The architecture of multiregion solvers in our previous work^44^ is transferred to the current CL model. In the model, pore and ionomer phase are integrated into one region and carbon is assigned to the other region. Since the transfer of electrons in Pt is not considered, Pt particles are removed from the computation, while the interface created between Pt and the other two phases (carbon and ionomer) highlights the reaction interface in the CL. The parameters for various simulations are listed in Table S1. The governing equations are discretized by second-order schemes. All simulations were implemented in parallel by using 128 processors (160 Intel Xeon@2.53 GHz/processor) in parallel. Detailed computational cost is listed in Table S1.

### CL Surrogate Model

The CL surrogate model is a data-driven 3D CNN model which consists of a series of 3D convolutional layers and is trained on the 3D latent space generated by the 3D encoder, as shown in Figure 1c. The model architecture is shown in Figure S9. The labels for training the surrogate model are *J* of the high-dimensional CL nanostructures. *J* labels are calculated by the pore-scale CL model in Figure 1e. L1 loss function in Pytorch was employed as follows
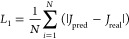
26where *J*_pred_ is the current density predicted by the 3D CNN and *J*_real_ the label current density. 400 3D latent were split into training and validation data sets at a ratio of 8:2. The batch size is 16. The model is trained 200 epochs with default Adam optimizer.

### PSO Algorithm

PSO is a typical bioinspired optimization algorithm which employs particle swarm to explore the design space. The location of particles is initialized randomly and then evolves based on the gradient of the objective function that is to maximize the *J* of the generated nanostructure. The location of particles corresponds to the Gaussian noise latent in the 3D generator of WGAN. The cognitive and social parameters are chosen as *c*_1_ = 2 and *c*_2_ = 2, respectively. The constant inertia weight *w* is 0.8. Ten particles were set for PSO. The initialized random numbers for particles were sampled from 0 to 1. Generally, PSO reaches steady convergence in 200–300 iterations regardless of the initial particle locations. The convergence is concluded when the optimal *J* remains the same in 200 iterations.

## Data Availability

The data set of Cryogenic-ET CL nanostructures is from ref (12). GDL and SOFC anode microstructures are from refs (27 and 28). The code is available from the corresponding author upon request.

## Supporting Information Available

Additional analyses for the reconstructed and generated CLs, SOFC anodes and GDLs; the prediction accuracy of the surrogate CNN model on the training data set; the stochastic algorithm for the CL generation; model architectures for the deep learning models involved in GLIDER; the model parameters for the multiphysics model of CLs (PDF)
