# Supplementary material for: Generative Artificial Intelligence for Designing Multi-Scale Hydrogen Fuel Cell Catalyst Layer Nanostructures
Source: ACS Nano. 2024 Jul 10;18(31):20504–17. doi: 10.1021/acsnano.4c04943 (PMC11308925; doi:10.1021/acsnano.4c04943)
Supplement: Supplementary file 1 — nn4c04943_si_001.pdf [file nn4c04943_si_001.pdf]

Supporting Information

Generative Artificial Intelligence for Designing Multi-Scale Hydrogen Fuel Cell Catalyst Layer Nanostructures

Zhiqiang Niu[b,*], Wanhui Zhao[f], Hao Deng[e], Lu Tian[b], Valerie J. Pinfield[d], Pingwen Ming[c], Yun Wang[a,*]

a Renewable Energy Resources Lab, Department of Mechanical and Aerospace Engineering, The University of California, Irvine, CA 92697, USA

b Department of Aeronautical and Automotive Engineering, Loughborough University, Loughborough, LE11 3TU, UK

c Clean Energy Automotive Engineering Centre, School of Automotive Studies, Tongji University, Shanghai, 201804, China

d Department of Chemical Engineering, Loughborough University, Loughborough, LE11 3TU, UK

e Shanghai Hydrogen Propulsion Technology Co., Ltd., Shanghai, 201800, China

f College of Aeronautical Engineering, Civil Aviation University of China, Tianjin, 300300, China

Corresponding author emails: z.niu@lboro.ac.uk (Z. Niu), yunw@uci.edu (Y. Wang).

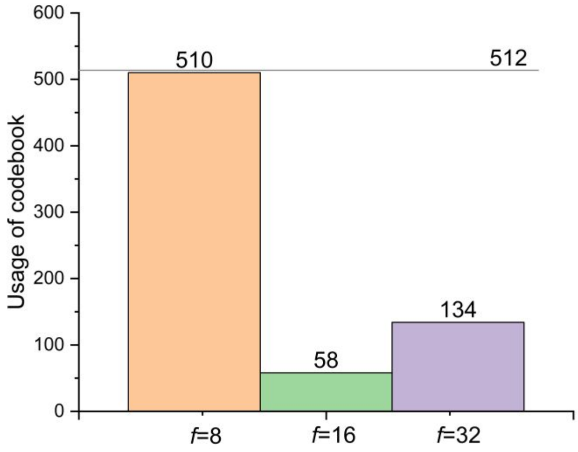
Usage of codebook
600
500
400
300
200
100
0
510
512
58
134
f=8
f=16
f=32

(a)

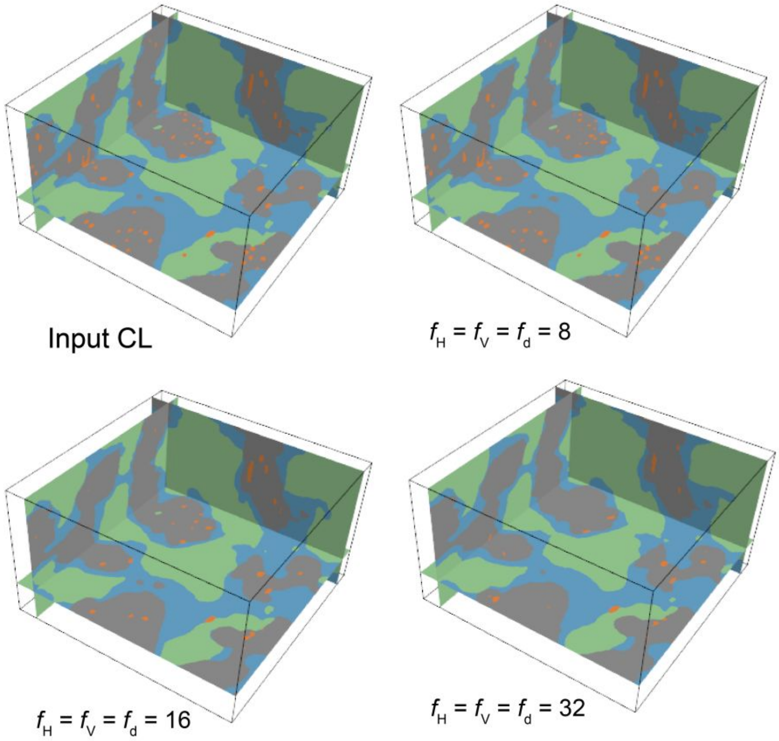
Input CL
$f_H = f_V = f_d = 8$
$f_H = f_V = f_d = 16$
$f_H = f_V = f_d = 32$

(b)

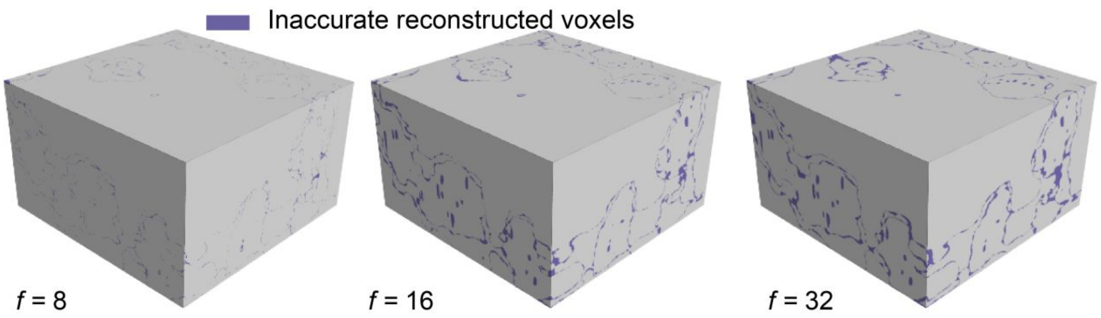
Inaccurate reconstructed voxels
f = 8
f = 16
f = 32

(c)

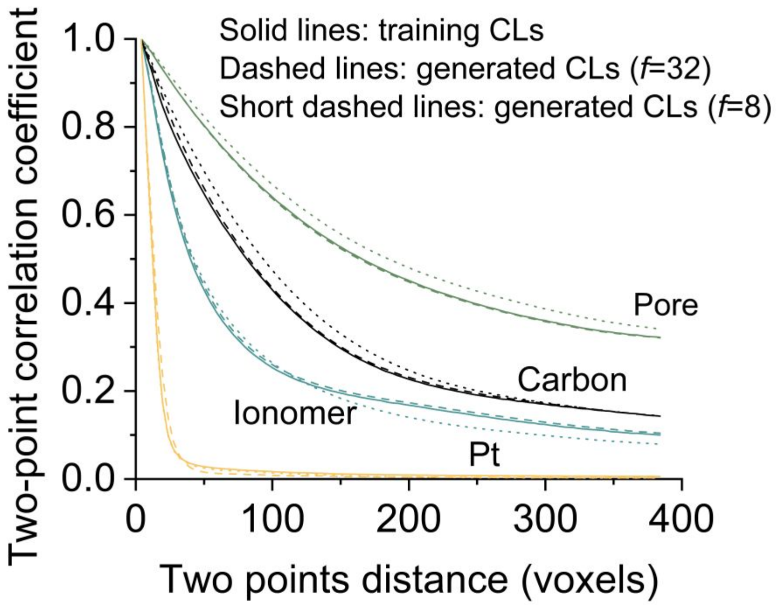

(d)

Supporting Figure S1 (a) The number of used vector labels in 100 test CL samples under three compression ratios *f* =8, 16 and 32. (b) Slice view of the input and reconstructed CL nanostructures in multiple direction within three cases *f* =8, 16 and 32. Here, pore (green) regions are displayed for comprehensive comparison. (c) The 3D distributions of the CL reconstruction error for under three compression ratios. (d) Two-point correlation coefficient curves of the real CLs (statistics on 100 samples), and the generated CLs in the case *f* = 32 (statistics on 100 samples) and *f*= 8 (statistics on 3 samples due to the prohibitive generation cost). (Supporting Information Page 3)

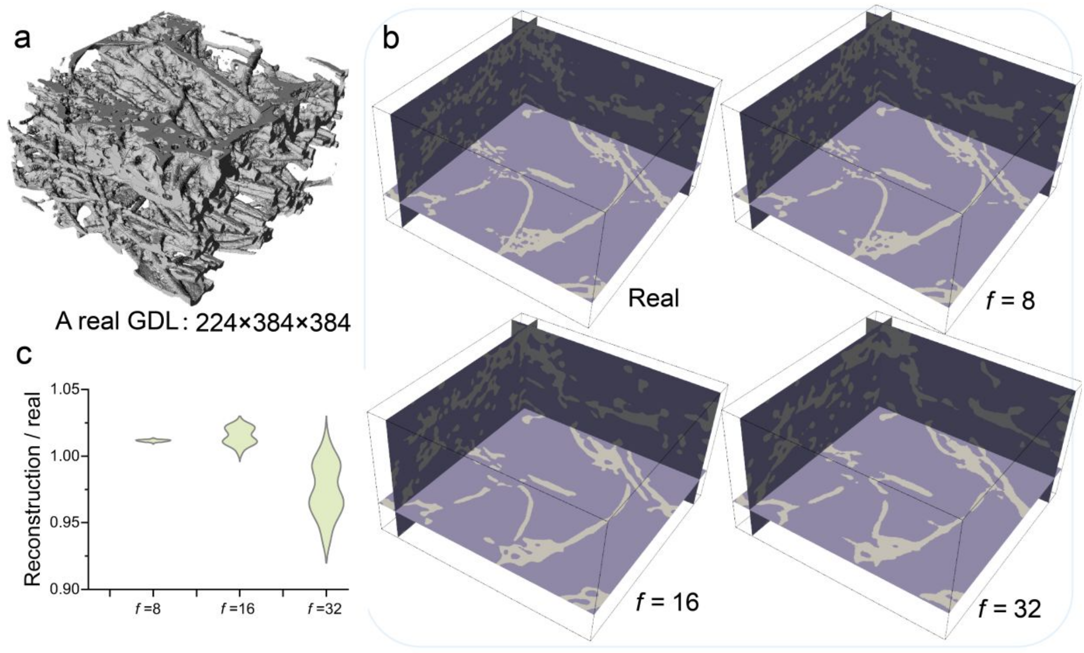

Supporting Figure S2 Evaluation of reconstruction loss when GLIDER compresses GDLs with various compression ratios f =8, 16 and 32. (a) 3D morphology of a real GDL with the

size 224×384×384 voxels (resolution 1.33 μm). (b) Real and reconstructed GDLs under various f values. (c) Quantitative analysis of the reconstructed fiber losses against real GDLs.

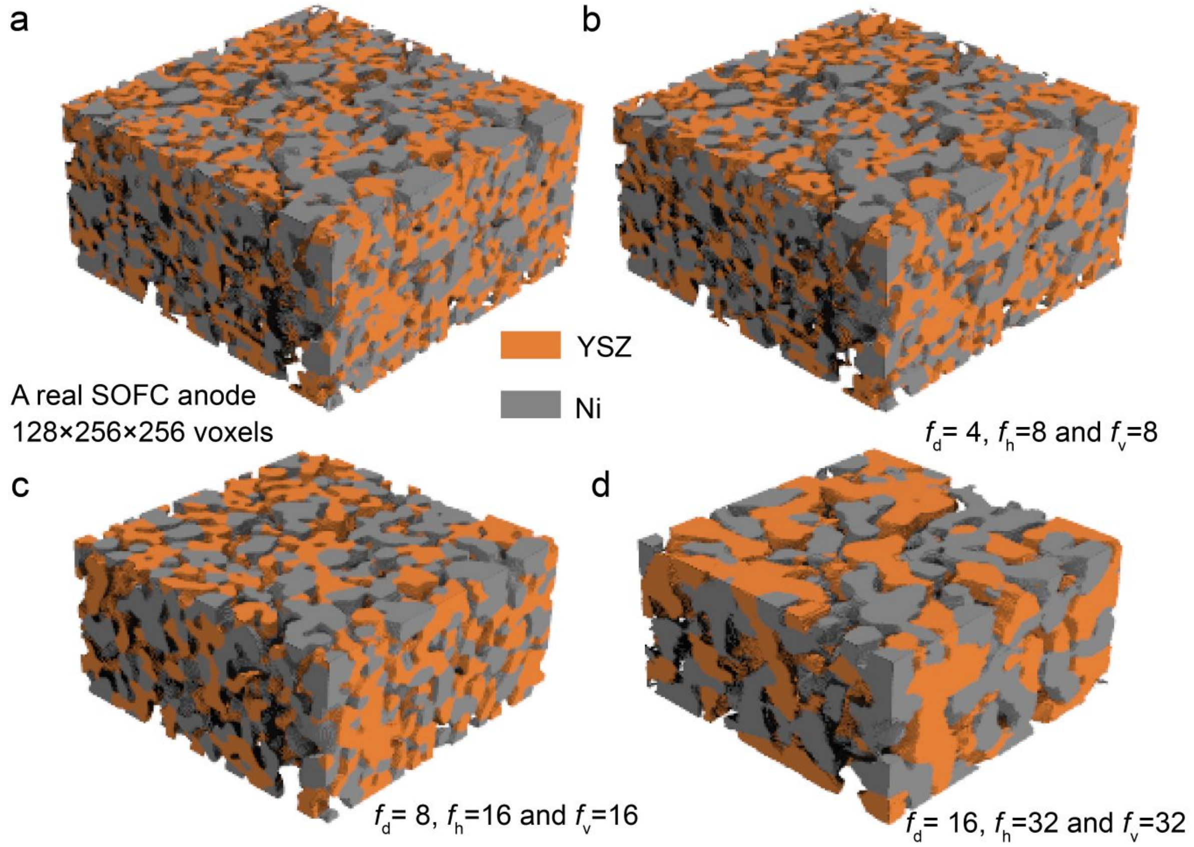

Supporting Figure S3 Comparison between the real (a) and the reconstructed SOFC anode microstructures with a size 128×256×256 voxels (resolution 65 nm) under three sets of compression condition: (b) f_d= 4, f_h=8 and f_v=8; (c) f_d= 8, f_h=16 and f_v=16; (d) f_d= 16, f_h=32 and f_v=32.

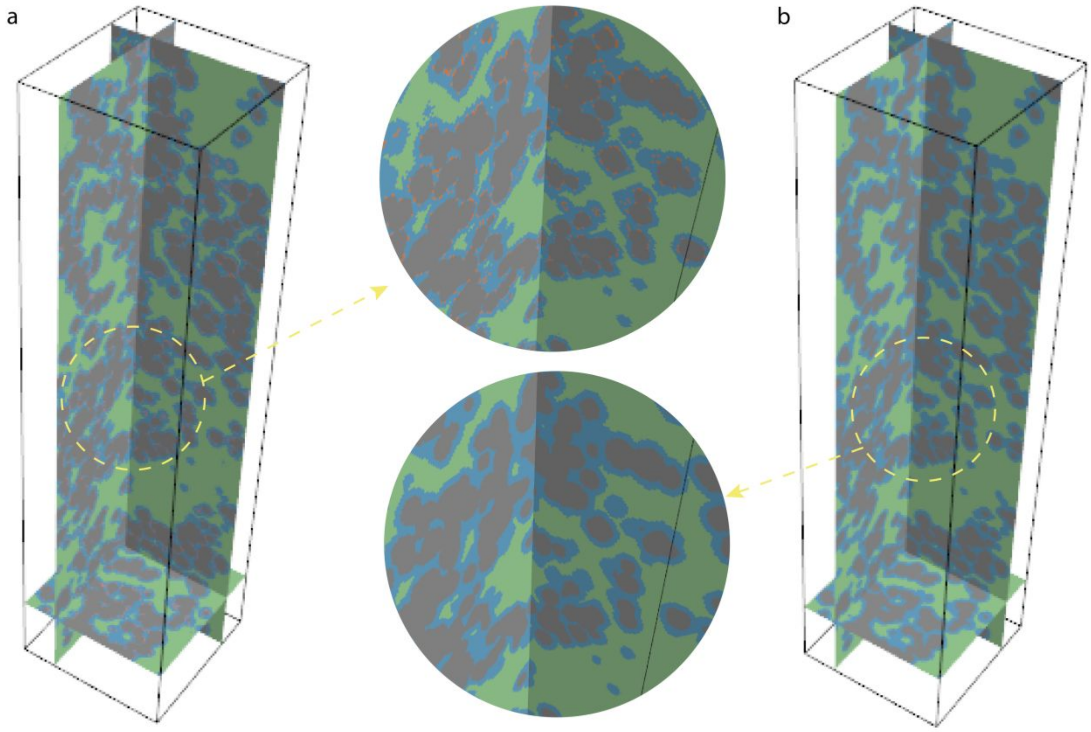

Supporting Figure S4 Reconstruction of CLs generated by the logic algorithm. The CL is resolved by 512×128×128 voxels (resolution 3.5 nm) under the compression ratio f_d= 4, f_h=16 and f_v=16. (a) training and (b) reconstructed CL.

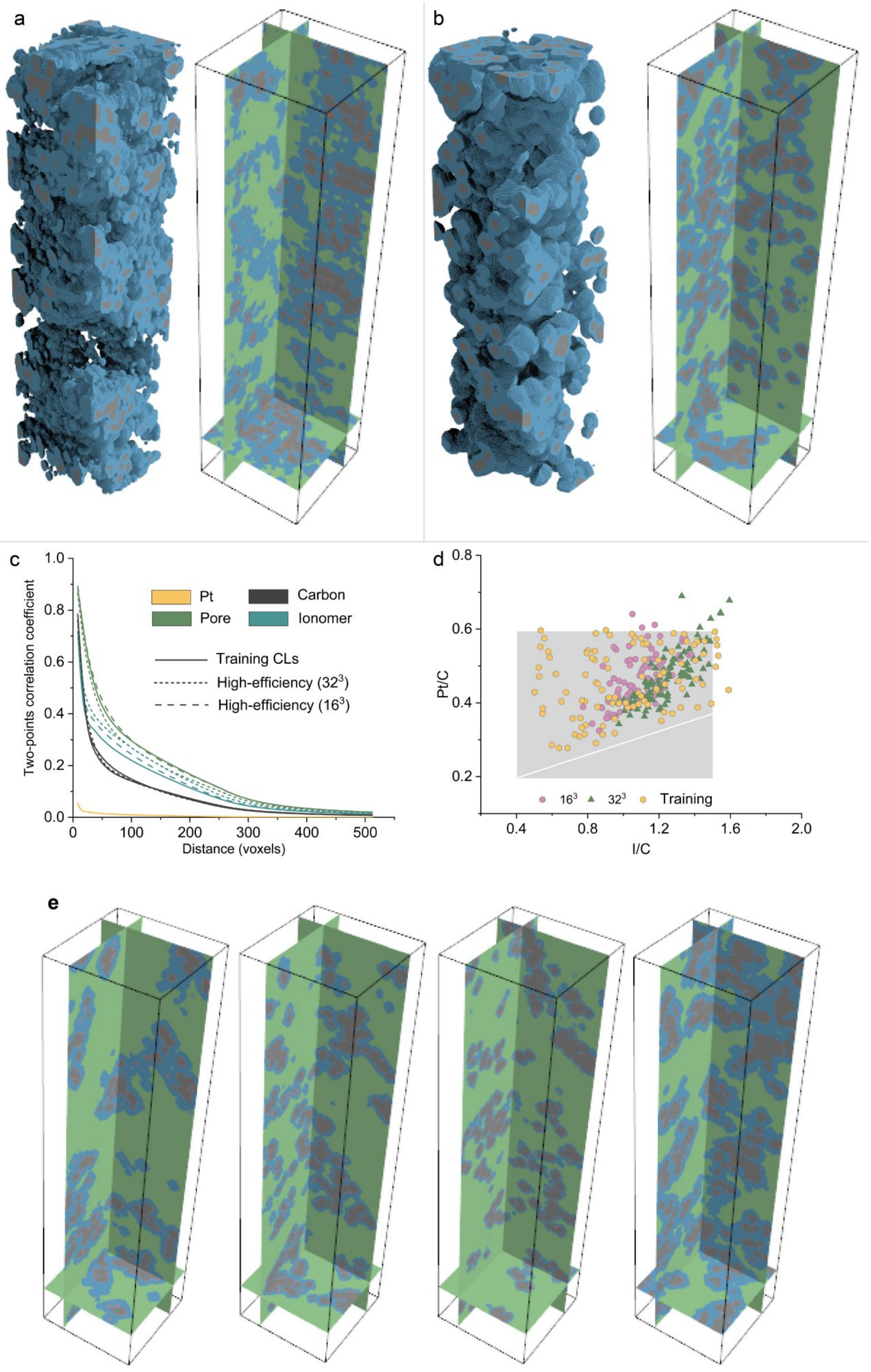

Supporting Figure S5 Generating CLs by transforming 32×32×32 latent under high-efficiency (a) and high-fidelity mode (b). (c) Two-point correlation coefficient curves averaged over 100

generated CLs based on two latent sizes under high-efficiency mode. (d) The generated distributions of Pt/C and I/C ratios of 100 CLs under high-fidelity mode. (e) Four representative CLs in the training dataset.

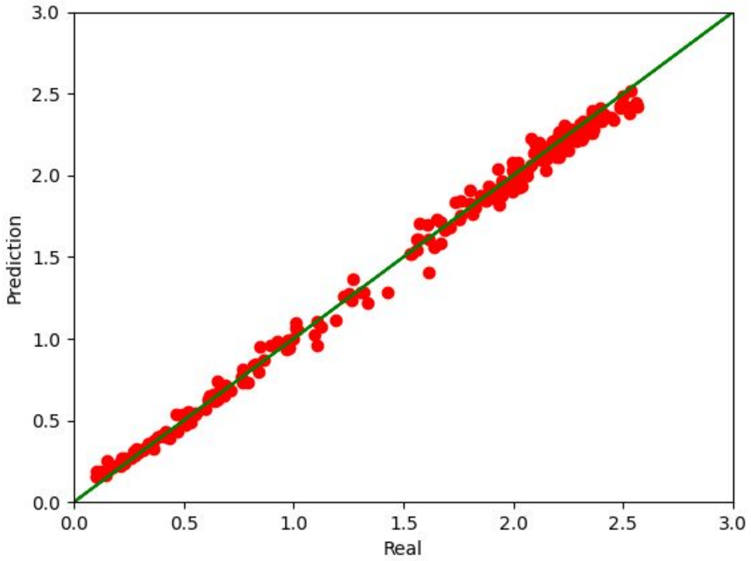

Figure S6 The accuracy of the surrogate CNN model for predicting *J* on the training dataset that is applied in Figure 6b.

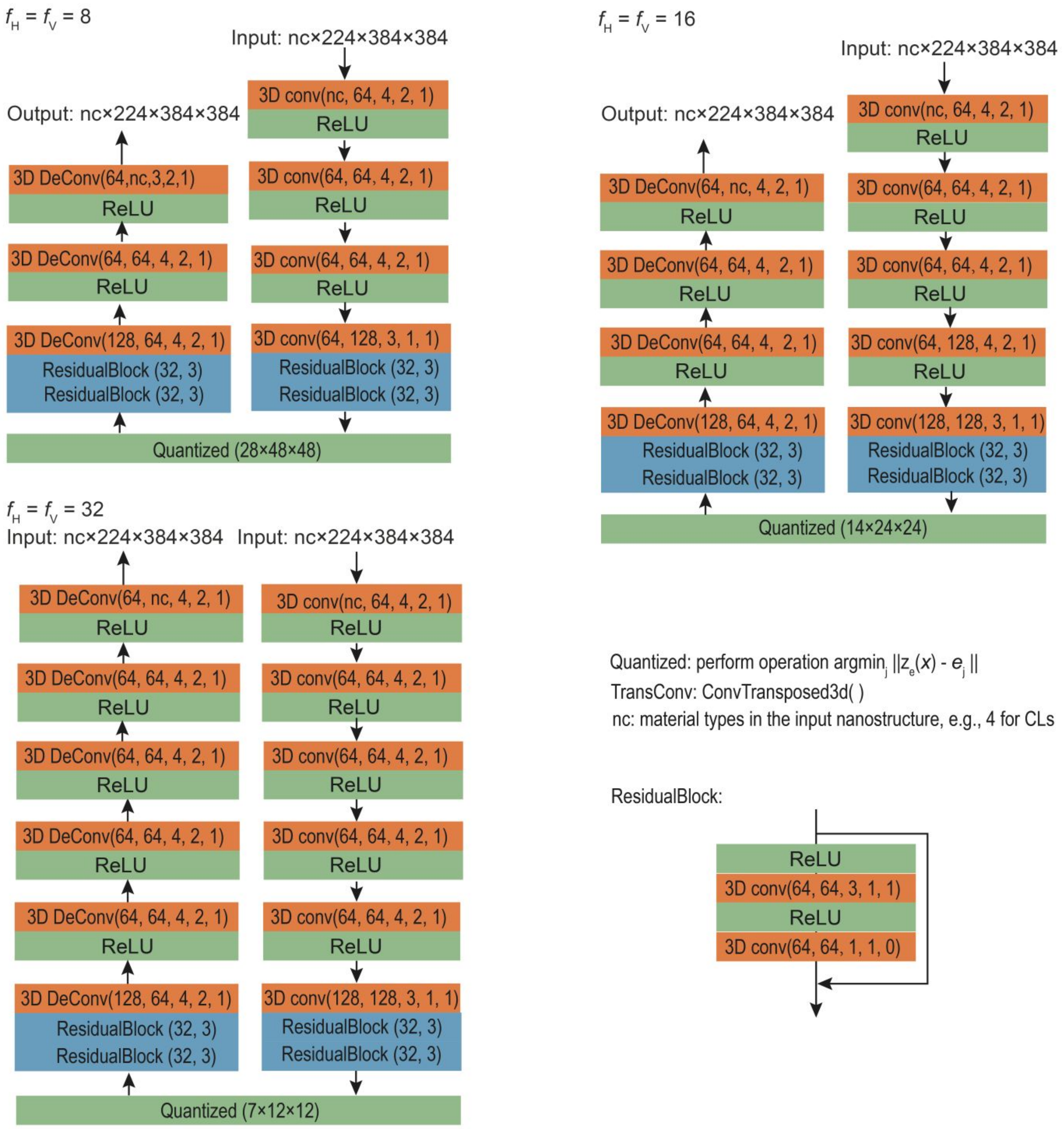

Supporting Figure S7 3D VQ-VAE model architecture. The modules in front of the Quantized layer composes the 3D encoder. The layers behind the Quantized layer make up the 3D decoder.

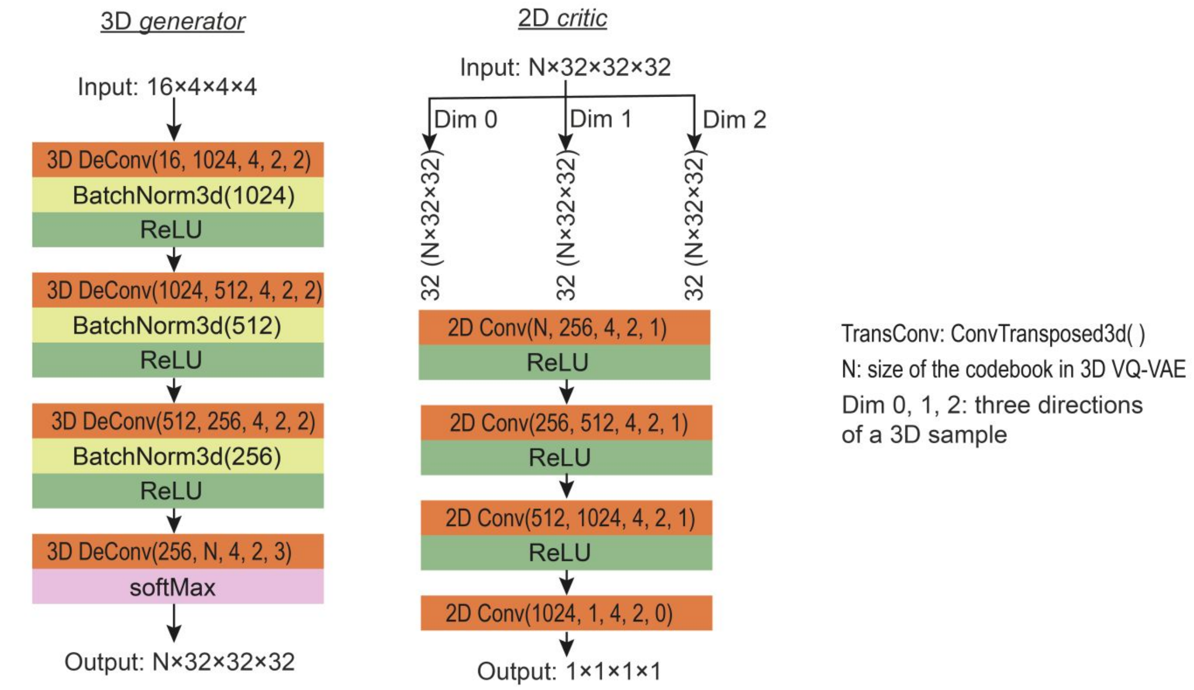

Supporting Figure S8 3D WGAN-GP model architecture.

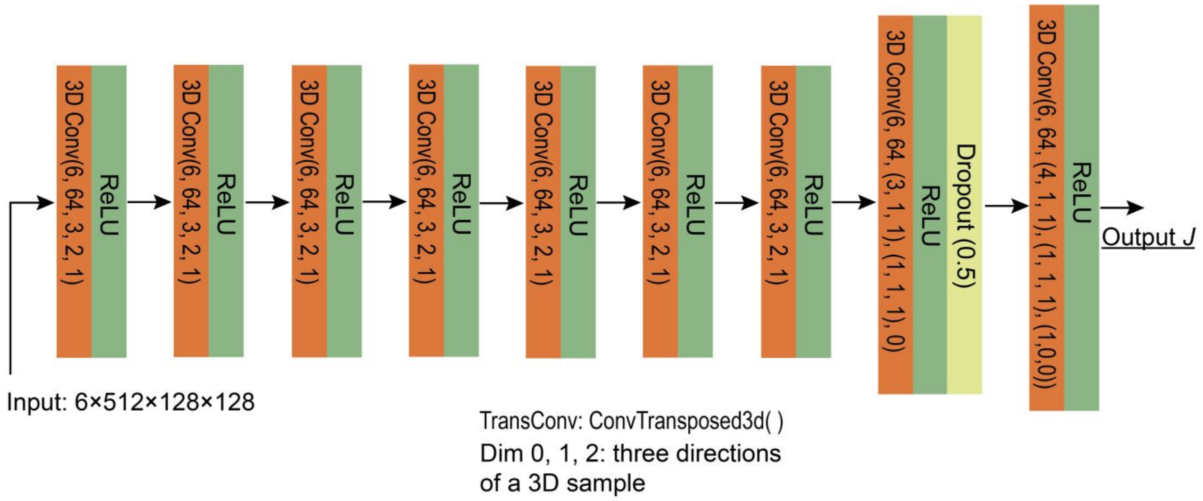

Supporting Figure S9 3D data-driven CNN architecture for the surrogate model of operating density *J*.

Supporting Note 1: The logic CL generation algorithm

The morphology of a typical CL of PEMFC is complicated due to the interaction among metallic Pt nanoparticles (2-3 nm) and amorphous carbon supports, covered by an ionomer film. To generate the digital nano structures of the CL, a stochastic algorithm is developed based on the following assumptions[6]:

1) Carbon supports are assumed as spheres with various diameters (30-70 nm).
2) Secondary pores in carbon supports are excluded.
3) Pt particles are assumed as single cubic boxes with a length of 3.5 nm.
4) Pt particles are assumed to attached to the carbon surface.

In the first step, a number of non-overlapped carbon spheres are added to the domain. Then, overlapped carbon spheres are further added until the volume fraction of carbon reaches the target. Subsequentially, Pt particles are attached to the carbon surface randomly until meeting the target Pt loading. The last step is to add ionomer to the above Pt/C nanostructures. The neighbor cells of Pt, carbon and ionomer are randomly assigned as ionomer until the calculated I/C reaches the target. The material properties employed in the algorithm are listed in Table 1. The Python code implemented for the algorithm is available in the section 'Data and code availability'.

During the logic CL generation with varying CL thickness, the volume fraction of each component in CLs is calculated as below:

$$\varepsilon_{\mathrm{Pt}} = (1-\varepsilon)\frac{\theta_{\mathrm{Pt}}/\rho_{\mathrm{Pt}}}{\theta_{\mathrm{I}}/\rho_{\mathrm{I}} + (1-\theta_{\mathrm{Pt}})/\rho_{\mathrm{carbon}} + \theta_{\mathrm{Pt}}/\rho_{\mathrm{Pt}}} \quad (1)$$

$$\varepsilon_{\mathrm{I}} = (1-\varepsilon)\frac{\theta_{\mathrm{I}}/\rho_{\mathrm{I}}}{\theta_{\mathrm{I}}/\rho_{\mathrm{I}} + (1-\theta_{\mathrm{Pt}})/\rho_{\mathrm{carbon}} + \theta_{\mathrm{Pt}}/\rho_{\mathrm{Pt}}} \quad (2)$$

$$\varepsilon_{\mathrm{carbon}} = (1-\varepsilon)\frac{(1-\theta_{\mathrm{Pt}})/\rho_{\mathrm{carbon}}}{\theta_{\mathrm{I}}/\rho_{\mathrm{I}} + (1-\theta_{\mathrm{Pt}})/\rho_{\mathrm{carbon}} + \theta_{\mathrm{Pt}}/\rho_{\mathrm{Pt}}} \quad (3)$$

The CL thickness (m) is calculated as follows:

$$L = \frac{\gamma_{\mathrm{Pt}}}{\varepsilon_{\mathrm{Pt}}\rho_{\mathrm{Pt}}} \quad (4)$$

where γ_{Pt} (mg cm^{-2}) denotes the Pt loading, set as 0.1 and 0.2, respectively, for model validation.

Regarding the logic CL generation with fixed thickness, the CL thickness L and Pt loading γ_{Pt} were set as 1.792 μm and 0.05 mg cm^{-2}, respectively. The volume fraction of Pt was calculated firstly based on the total Pt mass and Pt density. Then, the volume fraction of carbon, ionomer and pore were resolved according to Eqn. (1)-(3). To prepare the training CLs for optimization of the Pt/C and I/C ratios, 600 CLs were generated by randomly assigning Pt/C and I/C ratios from the range 0.2-0.6 and 0.5-1.6, respectively.

Table S1 Parameters of the pore-scale CL model.

Parameters	Case 1 (validation)	Case 2 (validation)	Case 3 (training)
**Pt loading** (mg cm^{-2})	0.1	0.2	0.05
**Pt/C**	0.4		0.2-0.6
**I/C**	0.507		0.5-1.6
**Porosity**	0.4		-
$\boldsymbol{\rho}_{\mathbf{carbon}}$ (kg m^{-3})		1800	
$\boldsymbol{\rho}_{\mathbf{Pt}}$ (kg m^{-3})		21450	
$\boldsymbol{\rho}_{\mathbf{ionomer}}$ (kg m^{-3})		2000	
$\boldsymbol{\alpha}$		0.5	
$\boldsymbol{J}_{\mathbf{ref}}$ (A m^{-2})		4×10^{-3}	
λ	14	22	14
$\boldsymbol{k}_{\mathbf{ele}}$ (S m^{-1})		5000	
$\boldsymbol{\tau}$		2	
$\boldsymbol{\tau_c}$		1×10^{5}	
$\boldsymbol{V}_{\mathbf{OCV}}$ (V)	0.97	0.98	0.97
$\boldsymbol{R}_{\mathbf{CL}}$ (s m^{-1})		30	
$\boldsymbol{R}_{\mathbf{t}}$ ($\boldsymbol{\Omega}$ $\mathbf{m^2}$)		9.3×10^{-6}	
**Mesh size**	742×144×144	1443×144×144	534×128×128
**Mesh resolution** (nm)		3.5	
**Computational cost** (CPU hours)	120	220	80
